# The Role of Methylation in the Intrinsic Dynamics of B- and Z-DNA

**DOI:** 10.1371/journal.pone.0035558

**Published:** 2012-04-17

**Authors:** Nuri A. Temiz, Duncan E. Donohue, Albino Bacolla, Brian T. Luke, Jack R. Collins

**Affiliations:** 1 In Silico Research Centers of Excellence, Advanced Biomedical Computing Center, Information Systems Program, SAIC-Frederick Inc., Frederick National Laboratory for Cancer Research, Frederick, Maryland, United States of America; 2 Department of Molecular Carcinogenesis, The University of Texas MD Anderson Cancer Center, Smithville, Texas, United States of America; Institut Pasteur, France

## Abstract

Methylation of cytosine at the 5-carbon position (5mC) is observed in both prokaryotes and eukaryotes. In humans, DNA methylation at CpG sites plays an important role in gene regulation and has been implicated in development, gene silencing, and cancer. In addition, the CpG dinucleotide is a known hot spot for pathologic mutations genome-wide. CpG tracts may adopt left-handed Z-DNA conformations, which have also been implicated in gene regulation and genomic instability. Methylation facilitates this B-Z transition but the underlying mechanism remains unclear. Herein, four structural models of the dinucleotide d(GC)_5_ repeat sequence in B-, methylated B-, Z-, and methylated Z-DNA forms were constructed and an aggregate 100 nanoseconds of molecular dynamics simulations in explicit solvent under physiological conditions was performed for each model. Both unmethylated and methylated B-DNA were found to be more flexible than Z-DNA. However, methylation significantly destabilized the BII, relative to the BI, state through the Gp5mC steps. In addition, methylation decreased the free energy difference between B- and Z-DNA. Comparisons of α/γ backbone torsional angles showed that torsional states changed marginally upon methylation for B-DNA, and Z-DNA. Methylation-induced conformational changes and lower energy differences may contribute to the transition to Z-DNA by methylated, over unmethylated, B-DNA and may be a contributing factor to biological function.

## Introduction

DNA methylation is one of the main epigenetic modifications contributing to gene regulation and a considerable amount of scientific effort has been devoted to understanding the mechanisms, roles, and effects of methylation in healthy and diseased states [1]. DNA methylation has been reported to play a role in development [2], gene silencing [3], and carcinogenesis [4]. In addition, the CpG dinucleotide, which represents the main target for methylation in humans, is a known hot spot for pathological mutations [5], [6].

**Figure 1:**
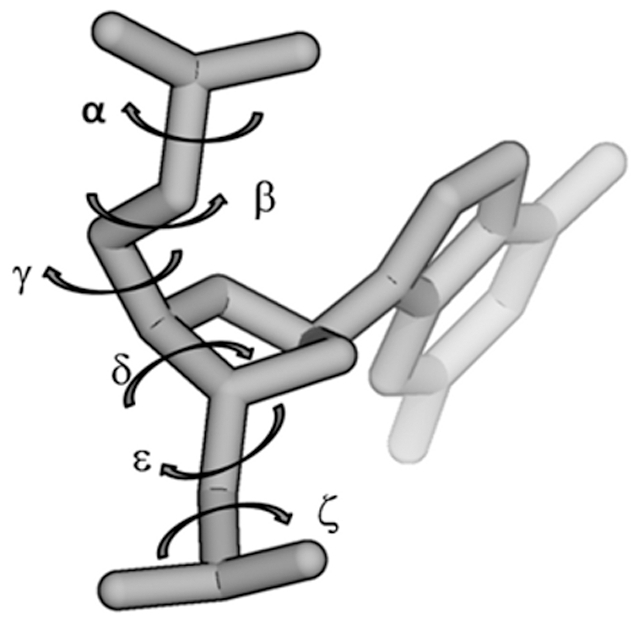
Schematics of torsional angles in a nucleotide phosphate backbone. α and γ angles determine canonical/non-canonical backbone conformations; ε and ζ define BI and BII sub-states of B-DNA.

DNA has a flexible backbone structure characterized by fluctuations in the torsional angles α, γ, ε, ζ, and sugar pucker (δ) (See Figure 1). The α, γ angles are associated with canonical/non-canonical backbone states and ε, ζ are associated with BI and BII substates. B-DNA has been observed to prefer the BI state over the BII state in crystals and solution [7], [8]. These two states are defined by the backbone torsional angles ε and ζ (Figure 1), where ε−ζ<0 defines the BI state and ε−ζ>0 defines the BII state. These conformational ensembles are important since they play a direct role in shaping the DNA backbone, and consequently underlie protein-DNA recognition [7], [8].

Most repetitive DNA sequences can assume different structures apart from canonical B-DNA, including cruciforms, triplexes, hairpins, quadruplexes and Z-DNA [9], [10], [11]. These “non-canonical” structural forms have been implicated in genomic instability and disease [12]. Z-DNA is a left-handed helical structure where the alternating purine (generally guanine) and pyrimidine (generally cytosine) base pairs form a zigzagging pattern [13]. The transition from right-handed B-DNA to left-handed Z-DNA is accompanied by a shift from the *anti* to the *syn* conformation of the alternating purines and has been shown to involve base extrusion at both B-Z junctions [14], [15]. Although both unmethylated and methylated CpG runs in B-DNA can switch to Z-DNA forms, methylation greatly favors this transition under physiological salt concentrations [16], [17], [18] or minute negative supercoiling [19].

Molecular dynamics (MD) simulations have been used extensively to study DNA structure and dynamics [20], [21], [22]. With the emergence of improved force fields [23], [24] and increasing computer power, simulations of B-DNA in the microsecond timescales have been conducted [22], [23], [25], [26]. However, despite the large number of simulations available, only 10–15 ns simulations of methylated B-DNA [27], [28] or unmethylated Z-DNA have been reported [23]. Since a detailed understanding of the effects of CpG methylation on the dynamics of both B- and Z-DNA at the molecular level is still lacking, the factors underlying methylation-assisted B-to-Z DNA transition remain largely unknown.

In an attempt to bridge this knowledge gap, structural models of a d(GC•GC)_5_ repeat sequence were constructed in ideal B-, methylated B-, Z-, and methylated Z-DNA forms. Two 50 nanoseconds MD simulations in explicit solvent under physiological conditions were performed for each, reaching a 0.4 microsecond aggregate simulation time. An analysis of the trajectories was then performed to examine the effect of methylation on the BI/BII stability in B-DNA and the sampling of non-canonical α/γ backbone torsional states (g+/t (gauche+/trans), g−/t (gauche−/trans), and g+/g− (gauche+/gauche−)) during the B- and Z-DNA simulations. The results indicate that methylation lowered the free energy difference between B- and Z-DNA. This suggests a lower energetic barrier, which may help explain the more facile change to Z-DNA by the methylated, rather than unmethylated, B-DNA.

## Results

### B-factors and thermal flexibility

**Figure 2:**
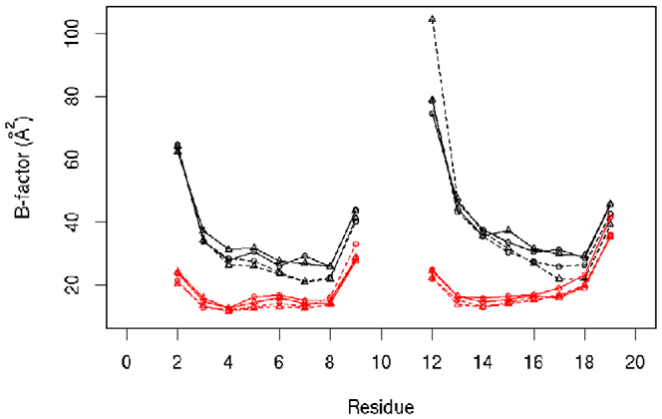
Theoretical B-factors for the four systems studied calculated from the simulations. *Black lines*, B-DNA simulations; *red lines*, Z-DNA simulations; *solid lines*, unmethylated DNA; *dahsed lines*, methylated DNA; *circles* and *tirangles*, independent simulation results for each system. Terminal bases not included in the calculatieon. Bases 11 to 20 form the complementary strand.

In this study, the effects of cytosine methylation on DNA dynamics are examined, including flexibility, base pair step parameters, base pair geometry and backbone torsion angles (Figure 1) in both the right handed canonical B form, and the left-handed non-canonical Z form. The flexibility of the structures is analyzed from the calculated B-factors by estimating the thermal mobility for each system (Figure 2) over the whole trajectory using the average structure of each trajectory as the reference. Note that terminal bases (residues 1, 10, 11 and 20) are excluded from the analysis. In addition, both methylated and unmethylated B-DNA display higher B-factors than Z-DNA (compare black, B-DNA, with red, Z-DNA, lines). Methylation is not seen to affect the fluctuations of Z-DNA, whereas it appears to slightly lower the B-factors in B-DNA (compare solid and dashed lines for B-DNA (black) and Z-DNA (red)).

The average structures of the heavy atoms of non-terminal bases obtained from the trajectories are used to calculate root mean square deviations (RMSD) (Figure S1). The results show that the two independent runs for B- and 5mCB-DNA yield very similar values (compare the black with red lines). A detailed analysis of the B-factors and RMSD values indicates that Z-DNA simulations (Figure S1C and D) have slightly lower values (∼1.0–1.3 Å (0.1 nm) RMSD) than B-DNA simulations (∼1.5–1.8 Å (0.15 nm)) (Figure S1A and B), implying that Z-DNA is overall less mobile than B-DNA.

### Effects of CpG methylation on B-DNA base pair and base pair step geometry

#### Base pair geometry

The sequence averaged conformational parameters from the eight independent simulations for base pairs (Table S1), base pair steps (Table S2), helix (Table S3) and backbone torsion angles (Table S4) show that, overall, the B-DNA simulations yield similar values to those recently reported by the Ascona B-DNA consortium [22] and those seen in crystal structures (Table S5). There is a marginal difference (1 degree) between the experimental averages and the simulations in the base pair parameter buckle (Tables S1 and S5). The distributions of individual geometric parameters from representative trajectories for B and 5mCB-DNA are displayed in Figures S2, S3, S4. Inspection of the data (Figure S2) indicates that base pair parameters for B and 5mCB-DNA form rather similar distributions and fall within one standard deviation of the crystallographically observed means (Table S5). Methylation does not appear to cause any large effect on the Watson-Crick base pairing in B-DNA as seen from base pair parameters shear, stretch, and stagger (Table S1). Nevertheless, the differences observed for buckle, propeller, opening, shear, stretch and stagger between the unmethylated and methylated B-DNA simulations (Table S1 and Figure S2), although marginal, are statistically significant, with -log(P) values of 16 (two sample paired t-test). This significance is likely revealed by the large number of data points used (1.6 million). Likewise, no differences are observed in base pair parameters when C•G and G•C base pairs were separated (data not shown).

#### Base pair step geometry

Base pair step parameters are within one standard deviation of the experimentally observed values (vertical dashed lines in Figure S3). The differences from the crystallographic data are in slide, roll and twist. Slide was positive (0.19 Å) in the crystallographic data but is negative (with values of −0.3 and −0.44 Å) in these simulations. The experimental mean of roll was 0.61°, whereas it is around 4° in the simulations. Finally, twist was 36° in the crystallographic data, but 33° in the simulations, similar to the value reported by the Ascona B-DNA Consortium [22]. Except for shift (P value = 0.11), the geometrically confined differences observed between the methylated and unmethylated simulations (Table S2) are also statistically significant, with –log(P) values of 16. The reason for the differences noted between methylated and unmethylated base pair step parameters becomes clear when the distributions for the GpC and CpG steps are separated (Figure S4). In this case, methylation is seen to narrow the base pair step distributions shift, tilt, roll, and to shift the distributions of twist and slide for the GpC steps relative to unmethylated B-DNA. On the other hand, the CpG steps are minimally affected, with the exception of slide.

#### Z-DNA base pair and base pair step geometry

As with B-DNA, the sequence averaged conformational parameters for the Z- and 5mCZ-DNA forms are similar on a gross scale (Tables S1, S2, S3, S4 and Figures S5, S6, S7, S8). However, statistically significant differences (−log(P) = 16) are noticed on a finer scale upon methylation for every geometric parameter, except for shear. Thus, the mean angle of base pair propeller decreased from 0.19 and −0.14 in Z-DNA to −0.50 and −0.28 in 5mCZ-DNA in the four independent simulations (Table S1). Similarly, base pair opening increased from 0.08 to 0.57 degrees upon methylation, whereas the standard deviation and the range of opening decreased (Table S1). Finally, in both independent runs, methylation increased the means of base pair step parameters tilt and roll, and decreased the standard deviations of tilt and roll with respect to the unmethylated simulations (Table S2). Interestingly, inspection of the distributions of the geometric parameters (Figures S5, S6, S7, S8) indicates that the mean buckle of the G•C base pairs decreased from −3 to −8 degrees, whereas that of the C•G base pairs increased from 3 to 8 degrees upon methylation (Figure S6). A similar shift is also observed in the distribution of the base pair step parameter rise (Figure S8), where the GpC step shifts to the left while the CpG step shifts to the right. Finally, the zigzagging nature of Z-DNA (CpG vs. GpC steps) results in bimodal distributions for the base pair step parameters slide and twist. In summary, methylation is seen to constrain fluctuations for a number of geometric parameters in Z-DNA.

#### B-DNA backbone dynamics

The effects of methylation on DNA backbone torsional angles and puckering are also analyzed. Figure S9 shows the representative density distributions of DNA backbone torsional angles from the B- and 5mCB-DNA simulations. Methylation modifies the distributions of the torsional angles δ, ε, and ζ to a certain extent. The α/γ and ε/ζ dynamics (Figure 1) will be discussed in more detail below. With regards to the puckering of the sugar backbone, Figure S10 shows the phase and amplitude distributions of the sugar pucker, as well as the distributions of torsional angles δ and χ. Overall, the χ angle and amplitude distributions display very similar profiles for the methylated and unmethylated forms. Minor differences are noted for the distributions of δ (Figure 1) and the phase of the sugar, which are attributed for the most part to a shift in the populations of C2′ Endo (from 35% in unmethylated B-DNA to 30% in methylated B-DNA) and O1′ Endo (from 16% in unmethylated B-DNA to 21% in methylated B-DNA) (Table S6). All four puckering parameters are in close agreement with the previously reported MD simulation averages [22]. In summary, methylation resulted in minute changes in sugar pucker dynamics for the B-DNA backbone.

#### Z-DNA backbone dynamics

In Z-DNA, changes are observed upon methylation in the distributions of the backbone angles α, γ, ε and δ (Figure 1), whereas no changes are detected for the β and ζ distributions (Figure S11). The zigzagging nature of Z-DNA results in two separate distributions for cytosine and guanine bases. Thus, the χ angle exists in *syn* conformation in guanine bases, whereas it is in the *anti* conformation in cytosines (Figure S12). Methylation also affects the distributions of the torsional angle δ for the guanine backbones.

#### BI–BII states

**Figure 3:**
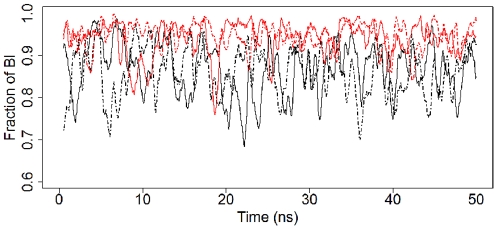
Time evolution of the fraction of BI states for B-DNA and 5mCB-DNA. The fraction of nucleotides in BI (ε/ζ) conformation are shown as a function of time; *black*, unmethylated B-DNA; *red*, methylated 5mCB-DNA; *solid* and *dashed lines*, two independent 50 ns simulations. The plots are smoothed using a 500 ps sliding window.

Crystallographic analyses have shown that canonical B-DNA comprises two conformational sub-states, BI and BII [7]. In the BI state, which is more common, the backbone phosphate adopts a rather symmetric position between the major and minor grooves, whereas in the BII state the phosphate groups are closer to the minor groove as a result of coupled changes in the two dihedral angles ε and ζ (Figure 1). Indeed, in the BI state ε and ζ are in the t/g− conformation, whereas in the BII state they switch to g−/t. In the present study, ε−ζ<0 and ε−ζ>0 are used as the cut-offs between the BI and BII states, respectively. Figure 3 shows the time evolution of the fraction of nucleotides in the BI state (Panel A) for B- (black) and 5mCB-DNA (red). The simulations for B-DNA are on average 84% in the BI state and 16% in the BII state, in agreement with previously reported results from a one microsecond B-DNA simulation [25] and with X-ray and NMR measurements [29], [30]. The simulations for 5mCB-DNA, on the other hand, show a BI population of 92% and, correspondingly, a BII population of 8%. The results of the time evolution of ε−ζ for B- and 5mCB-DNA (Figures S13 and S14) and the cumulative averages of the BI states from the simulations (Figure S15, top panel) support the conclusion that methylation stabilizes the BI state [28]. Using a similar approach to Rauch et al. [28], the free energy profiles for the BI/BII transitions were calculated from the B- and 5mCB-DNA simulations. Since no major differences were seen between the two independent runs, for either the B- or 5mCB-DNA cases, the data for the reaction coordinate ε−ζ were combined, resulting in 1.6 million observations. These data were used to plot the histograms for the ε−ζ distributions using 18 degree bins (20 total bins). Assuming the simulations were long enough to cover the available angle space, the partition function Z was calculated and the free energy at each bin was obtained using G = −RTln(Z) [28].

**Figure 4:**
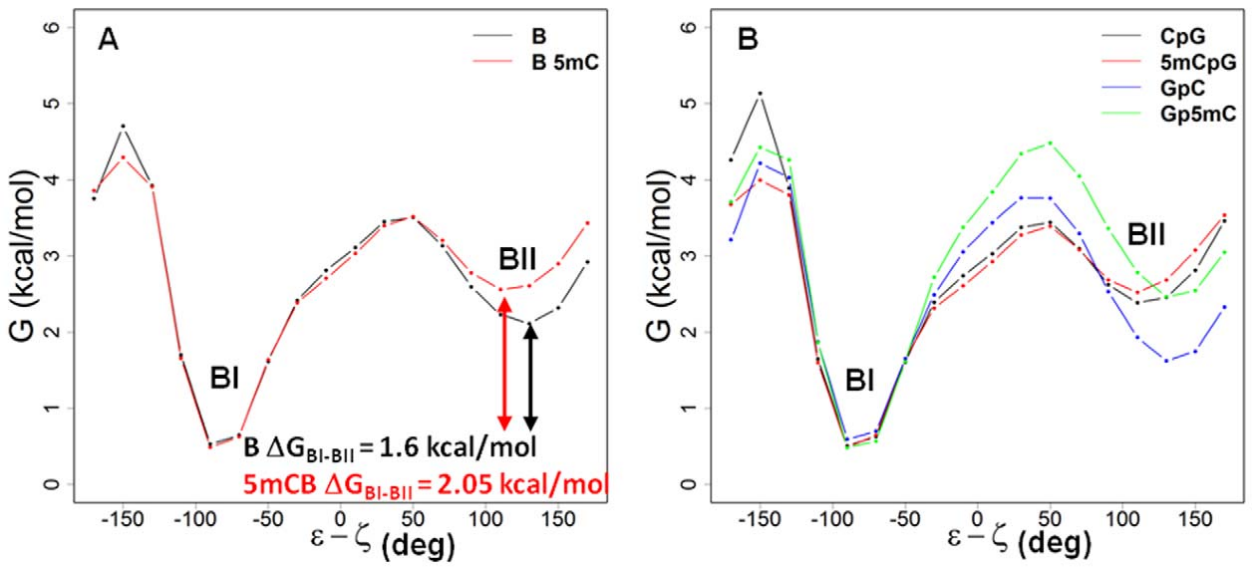
Relative free energy profiles across the ε−ζ reaction coordinates in B- and 5mCB-DNA. The plots show the changes in free energy (y-axis) across the ε−ζ coordinate range (x-axis) that define the BI and BII sub-states. (A) Overall relative free energy profiles for unmethylated B-DNA (*black*) and methylated 5mCB-DNA (*red*). (B) Relative free energy profiles for unmethylated and methylated CpG and GpC steps. *Black*, CpG steps of B-DNA; *red*, 5mCpG steps of 5mCB-DNA; *blue*, GpC steps of B-DNA; *green*, Gp5mC steps of 5mCB-DNA.

Figure 4A shows the overall free energy profiles for B-DNA (black) and 5mCB-DNA (red). The free energy differences between the BI and BII states (ΔG_BI–BII_) are 1.6 kcal/mol in B-DNA and 2.08 kcal/mol in 5mCB-DNA. Interestingly, when the free energy profiles for the CpG versus GpC steps are separated, an increase in ΔG_BI–BII_ from 1.02 kcal/mol (B-DNA) to 1.98 kcal/mol (5mCB-DNA) is revealed for the GpC steps, whereas no effect is observed for the CpG steps. (ΔG_BI–BII_ = 0.16 between B-DNA and 5mCB-DNA) (Figure 4B). Also, the barrier height of the BI–BII transition is virtually unchanged after methylation, so the BII-BI transition is 0.46 kcal/mol lower for 5mCB-DNA compared to unmethylated B-DNA. The differences observed in the free energies for the BI and BII states (Figure 4) are also documented by the average residence times in B- and 5mCB-DNA simulations. B-DNA simulations show an average of 216±395 ps residence time in the BI state and 28±36 ps residence time in BII. 5mCB-DNA simulations, on the other hand, display 153±518 ps residence time in the BI state and 10±21 ps in BII. The number of passages between BI and BII states decreases from around 6039–6099 to 4676–4934 upon methylation.

#### α/γ transitions in B-DNA

**Figure 5:**
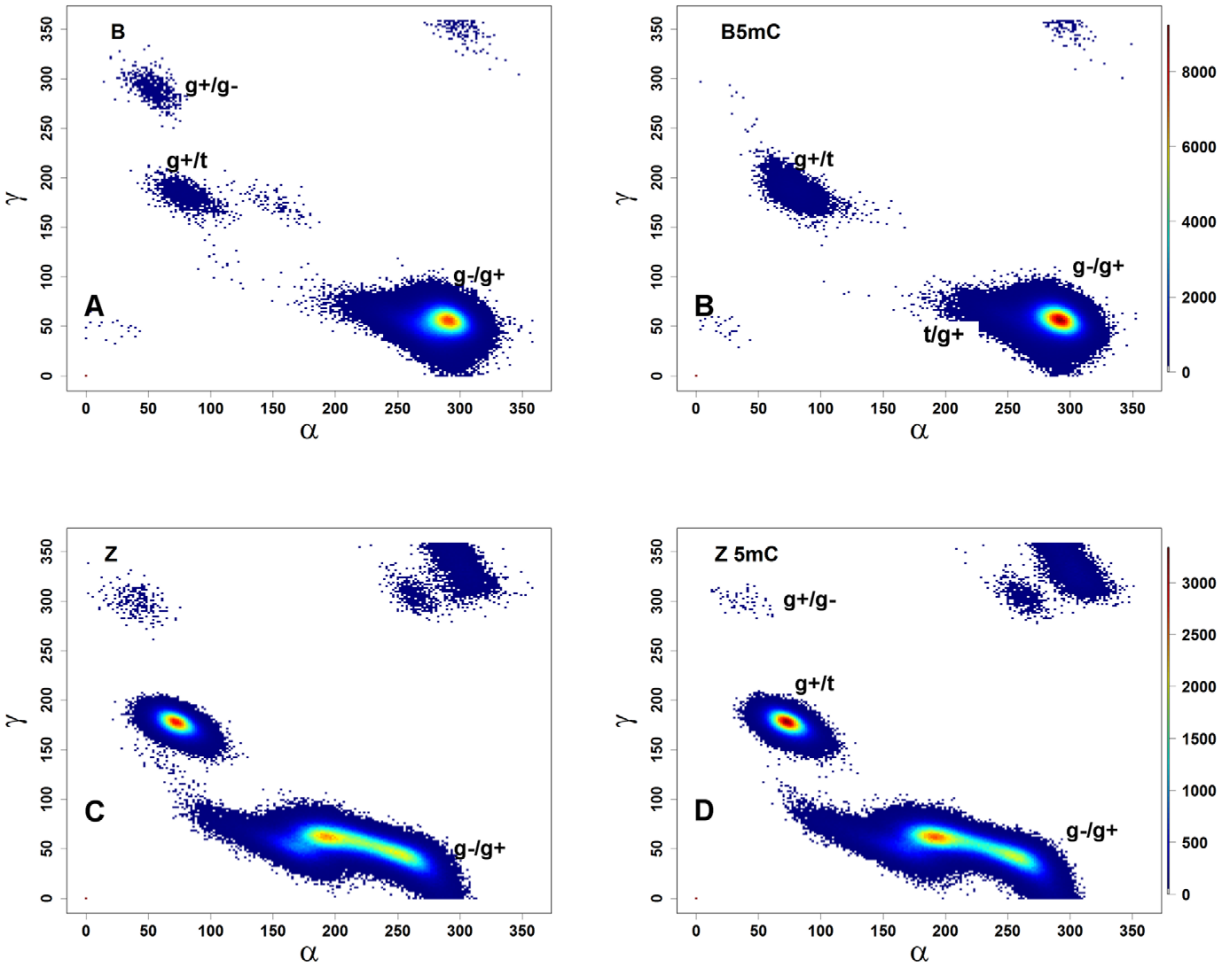
The α/γ distributions in B- and Z-DNA. **Landscape of the combined distributions of phosphate torsion angles along the α/γ space.** (A) B-DNA; (B) 5mCB-DNA; (C) Z-DNA; (D) 5mCZ-DNA. The plots are color-coded based on the density of points. The results from the two independent simulations for each state are combined, giving 1.6 million points. The color bars on panels B and D show the density values for B- (panels A and B) and Z-DNA (panels C and D) simulations.

**Figure 6:**
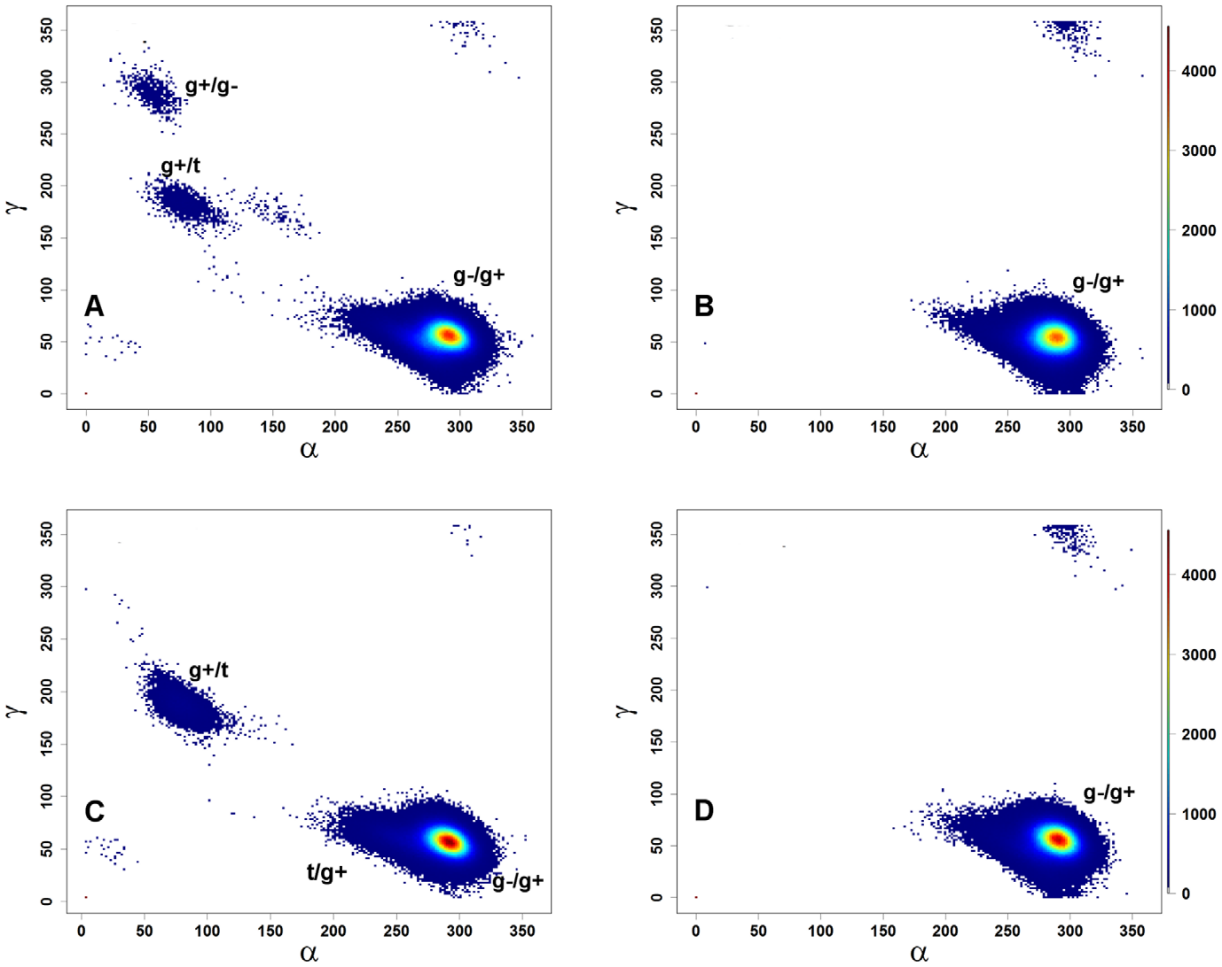
Scatter plots of α vs. γ for CpG and GpC steps. (A) GpC steps in B-DNA; (B) CpG steps in B-DNA; (C) Gp5mC steps in 5mCB-DNA; (D) 5mCpG steps in 5mCB-DNA. The plots are color-coded based on the density of points.

The effects of methylation on the BI/BII states suggest that methylation caused significant backbone torsional rearrangements. In solution, free B-DNA is mostly found in the canonical (g−/g+) α/γ states; in protein-DNA complexes, on the other hand, DNA exhibits a higher percentage (∼15%) of non-canonical states [31], [32], which are believed to assist protein-DNA interactions [31], [32]. Because the simulations used the *parmbsc0* parameter set [23], which corrects the non-canonical conformers of B-DNA, unmethylated B-DNA is seen to sample canonical conformations over 99% of the time (Figure 3B, black lines), as expected [25]. For the methylated 5mCB-DNA simulations (Figure 3B, red lines), all nucleotides sample canonical states similarly to unmethylated DNA (Figure S15, bottom panel). The distribution of the backbone torsions over the α/γ space is shown in Figure 5. Both B-DNA and 5mCB-DNA almost exclusively prefer the canonical (g−/g+) conformations (Figure 5A–B and Table S7) with the exception of g+/t (1% in 5mCB-DNA) and t/g+ space (1% in 5mCB-DNA). To address the question as to whether methylation affected α/γ state sampling to a similar extent for the two types of base pair steps (CpG and GpC), separate plots were generated for the α/γ states based on the CpG (α/γ angles of G) and GpC (α/γ angles of C) steps for B- and 5mCB-DNA simulations (Figure 6). In unmethylated B-DNA, the GpC steps sample the non-canonical conformations g+/g−, g+/t, and g+/t (less than 1% of simulation time, Figure 6A), whereas CpG steps spend all of the simulation time in canonical g−/g+ conformations (Figure 6B). Upon methylation, Gp5mC steps sample an increased number of g+/t (greater than 1% of simulation time, Figure 6C) and t/g+ states (Figure 6C). Thus, although methylated and unmethylated DNA spend most of the time in the canonical g−/g+ conformational state (Table S7), methylation causes the GpC step to sample an increased number of the non-canonical states g+/t and t/g+. In summary, GpC steps contribute to the difference in sampling of non-canonical conformers induced by methylation in 5mCB-DNA.

#### α/γ transitions In Z-DNA

Although on a gross scale the α/γ torsions show similar distributions in the Z- and 5mCZ-DNA simulations (Figure 5C and D), a number of differences are observed upon methylation. These include a shift from g−/g+ to t/g+ and an increase in the number of conformations in the g+/t state (Figure 5C and D). The increase in the g+/t populations could unambiguously be attributed to the GpC step, whereas the shift from g−/g+ to t/g+ is mainly caused by the CpG step (Figure S16). The α torsions consistently lost the *trans* conformations in the cytosine backbone (GpC steps) after methylation, thereby switching to g+ (Figure S16A and C). The γ torsions, on the other hand, lost g+ states and increased the *trans* population, thus compensating for the changes in the α torsion angle. In the CpG steps, the α torsions shift to *trans*, whereas the γ torsions remain unchanged (Figure S16B and D). In summary, methylation caused shifts in backbone torsional preferences in Z-DNA.

## Discussion

In this study, MD simulations are conducted to assess the role of methylation in the intrinsic dynamics of a 10 base paired d(GC•GC)_5_ repeat, both in the canonical B-DNA and non-canonical left-handed Z-DNA forms. The work is motivated by the critical role that 5mCpG methylation plays in human development and cancer [2], [3], [4] and by the fact that methylated d(GC•GC)_n_ sequences facilitate B- to Z-DNA structural transitions [17], [33] by mechanisms that are not fully understood, leading to genetic instability [10], [12].

Our results confirm that unmethylated B-DNA almost exclusively samples canonical α/γ states and methylation only marginally increases sampling of non-canonical states, particularly for the Gp5mCp steps. By contrast, unmethylated and methylated Z-DNA sample non-canonical conformations extensively (∼30–50% of the time) (Figure S15 and Table S7). It has been shown that Z-DNA exists in substates, ZI and ZII, using FT-IR spectroscopy [34]. ZI and ZII states are defined mainly by α and ζ torsional angles. In our simulations, we did not see any effect of methylation on the structure or free energy difference of these states (data not shown).

MM/PBSA [35], [36], [37] methods have been extensively used to study conformational stability of nucleic acids [36], [37], and ligand-DNA interactions [35]. Here, simple MM/PBSA analyses were performed to infer the free energies and stability of the systems studied (Table 1). To have a second estimate for configurational entropy we used ACCENT-MM [38], but 50 nanoseconds of simulation time was not sufficient for convergence for the B- and Z-DNA models (data not shown). Both methylated and unmethylated B-DNA are more stable than their Z-DNA counterparts. The calculated free energy difference between B- and Z-DNA is 21.6 kcal/mol and between methylated B- and Z-DNA is 14.4 kcal/mol. A recent targeted molecular dynamics study of a B-Z junction has reported a barrier of 13 kcal/mol and a free energy difference of 4.7 kcal/mol for a 10 base pair DNA sequence, proposing a sequential zipping mechanism for Z-DNA formation [39]. Although our numbers are higher than those of Lee et al. [39], they are close to the experimental free energy range for B-Z (12–17 kcal/mol) and methylated B-Z (9 kcal/mol) transitions [19], [40]. The differences in the calculated free energies are found to be statistically significant using a two sample t-test with -log(P)>23, implying that the B-Z transition barrier is lower for the methylated than the unmethylated system, in agreement with experimental observations [19], [40].

**Table 1:** MM/PBSA analysis of B, methylated B (BM), Z and methylated Z (ZM) DNA.

	B	SE_B_ 1	Z	SE_Z_ 1	ΔB-Z	BM	SE_BM_ 1	ZM	SE_ZM_ 1	ΔBM-ZM
**〈E_ELE_〉** 2	−777.4	1.15	−207.3	2.34	570.1	−880.0	5.49	−302.5	2.29	577.56
**〈E_VDW_〉**	−165.7	0.29	−192.2	0.30	−26.5	−172.1	0.29	−202.7	0.29	−30.59
**〈E_INT_〉**	932.7	0.64	968.4	0.63	35.7	974.2	0.63	1016.4	0.65	42.14
**〈E_MM_〉**	−10.4	1.19	568.8	2.34	579.3	−94.0	1.24	511.2	2.31	605.24
**〈E_PBSUR_〉**	22.9	0.01	21.0	0.01	−1.9	23.3	0.01	21.0	0.01	−2.26
**〈E_PBCAL_〉**	−4655.9	1.05	−5219.6	2.20	−563.6	−4627.9	1.13	−5226.0	2.18	−598.09
**〈E_PBSOL_〉**	−4633.0	1.05	−5198.5	2.20	−565.5	−4604.6	1.14	−5204.9	2.19	−600.34
**〈E_PBELE_〉**	−5433.4	0.38	−5426.9	0.43	6.5	−5524.0	0.40	−5528.4	0.40	−4.40
**〈E_PBTOT_〉**	−4643.4	0.59	−4629.7	0.61	**13.8**	−4698.6	0.58	−4693.7	0.65	**4.89**
**-T〈 S〉**	−524.6	0.21	−516.8	0.20	**7.9**	538.2	0.22	528.8	0.20	**9.46**
**〈G〉**	−5168.1	0.64	−5146.4	0.64	**21.6**	−5236.8	0.63	−5222.5	0.67	**14.36**

1 SE, standard error.

2 All energy values are in kcal/mol.

MD simulations have been extensively employed to study nucleic acids dynamics [41]. Although these analyses can now reach millisecond time scales [25], artifacts have been found in the Amber *parm99* force field in the form of extensive α/γ transitions [42], [43], [44]. The *Parmbsc0* force field [23] was introduced to correct for these artifacts. Thus, despite perceived limitations to large-scale conformational predictions [22], our results support the use of MD simulation studies as a means to predict DNA dynamics.

The overlapping conformational states by concerted phosphate backbone torsional angle switches observed in both methylated B- and unmethylated/methylated Z-DNA (Figure 6) agree with recent NMR [45] and single molecule fluorescence data [18].

In summary, MD simulations were used to further understand the role of cytosine methylation on both the canonical B-DNA and non-canonical left-handed Z-DNA structures. The results show that methylation lowers the free energy difference between B and Z-DNA resulting in the increased population of Z-DNA. We suggest that methylation-induced differences in the CpG and GpC steps' backbone dynamics may facilitate the initial step in the mechanism of B to Z transitions.

## Methods

### Model Building

Four structural models of 10 base paired d(GC•GC)_5_ repeats were built using the canonical B- and Z-DNA settings in w3DNA [46]. The cytosine bases of two model structures (one Z- and one B-DNA) were then manually methylated at the 5-carbon position using UCSF Chimera [47] at all positions except the terminal bases. Therefore, the procedure resulted in an overall 80% methylation of the cytosines (8 out of 10) simulating a hypermethylated state [48]. The model structures are named B, 5mCB, Z, and 5mCZ for B-DNA, methylated B-DNA, and Z-DNA and methylated Z-DNA, respectively.

### MD Simulations

MD simulations were performed using the AMBER 10 simulation package [49]. The AMBER *parm99* [24] with *parmbsc0* corrections [23] and TIP3P water molecules [50] were used to represent molecular interactions. Parameters for 5-methyl-cytosine (5mC) were taken from Rauch et al., 2005 [27], who also employed *parm99* [24]. Each system was neutralized with 18 Na^+^ ions and solvated with approximately 5000 water molecules for the B-DNA models and 5450 water molecules for the Z-DNA models in truncated octahedral boxes. Additional Na^+^ and Cl^−^ ions were added by randomly replacing water molecules, to bring the system to 150 mM salt concentration (34 and 32 ions to the Z-DNA and B-DNA models, respectively). The radial distribution functions of counterions around DNA for all simulations (data not shown) agree well with the previously published distributions [51], [52], [53]. A 10 Å cut-off was used for non-bonded interactions, along with the Particle Mesh Ewald [54] method. SHAKE [55] was used for hydrogen atoms. A 2 femtoseconds time step was used for the simulations. The systems were energy minimized and then heated to 300 K in 20 picoseconds (ps) at constant volume with 100 kcal/mol/Å^2^ harmonic restraints on all solute atoms. The harmonic restraints were then reduced to 50, 10, 5, and 1 kcal/mol/Å^2^ in 20 ps intervals, followed by 380 ps of unrestrained equilibration at constant pressure and temperature. Two independent 50 ns-long production runs were performed for each system starting with different initial velocities. The aggregate simulation time was 400 nanoseconds. Trajectories were analyzed using the MM/PBSA and PTRAJ modules of AMBER 10, as well as Curves+ [56], and custom R [57] scripts. To avoid end effects, the terminal base pairs were removed from the analyses of geometric parameters and torsional angles. The density distributions of the various parameters were calculated using the kernel density function of R, which estimates the probability density function of the variables.

### MM/PBSA analysis

Conformational free energies were calculated by the MM/PBSA method using the perl scripts available within the AMBER 10 [49] simulation package. Snapshots for the MM/PBSA analysis were extracted from all eight simulations in 100 ps intervals, yielding 500 snapshots per independent trajectory. Absolute free energies were calculated using the equation G = E_MM_+E_PB_+E_SA_−TS, where E_MM_ is the molecular mechanics energy, E_PB_ is Poisson Boltzmann energy, E_SA_ is the nonpolar solvation free energy and TS is the entropic contribution. E_SA_ was assumed to be proportional to the solvent accessible surface area (SA), i.e. E_SA_ = γSA+b, with the coefficients set to default γ = 0.00542 kcal/Å^2^ mol and b = 0.92 kcal/mol. The AMBER *nmode* program was used to estimate the vibrational entropies after one thousand step energy minimization [58]. The results were averaged over the 500 snapshots for each system.

## Supporting Information

Figure S1 Time evolution of root mean square deviation (RMSD) of the heavy atoms from their mean positions during the simulations. A. B-DNA B. 5mCB-DNA C. Z-DNA D. 5mCZ-DNA. Black and red lines represent the two independent runs for each system. The terminal bases are excluded.
(PNG)

Figure S2 Representative distributions of base pair parameters in B-DNA (black) and 5mCB-DNA (red) simulations. Vertical dashed lines indicate the mean ± one standard deviation of the crystallographically determined values (see Table S5). Top row x-axes (buckle, propel, opening) are in degrees and bottom row x-axes (shear, stretch, stagger) are in Angstroms. Y-axes show the densities.
(PNG)

Figure S3 Representative distributions of base pair step parameters in B-DNA (black) and 5mCB-DNA (red) simulations. Vertical dashed lines indicate the mean ± one standard deviation of the crystallographically determined values (see Table S5). Top row x-axes (shift, slide, rise) are in Angstroms and bottom row x-axes (tilt, roll, twist) are in degrees. Y-axes show the densities.
(PNG)

Figure S4 Representative distributions of base pair step parameters for GpC and CpG steps in B-DNA and 5mCB-DNA simulations. Black and green lines show the CpG steps, whereas red and blue lines show the GpC steps for the B-DNA and 5mCB-DNA simulations, respectively. Y-axes show the counts. X-axes are in Angstroms in the top row (shift, slide, rise) and degrees in the bottom row (tilt, roll, twist).
(PNG)

Figure S5 Representative distributions of base pair parameters in Z-DNA (black) and 5mCZ-DNA (red) simulations. Y-axes show the densities. X-axes are in degrees in the top row (buckle, propel, opening) and Angstroms in the bottom row (shear, stretch, stagger).
(PNG)

Figure S6 Representative distributions of base pair parameters for GpC and CpG steps in Z-DNA and 5mCZ-DNA simulations. Black and green lines show the CpG steps, whereas red and blue lines show the GpC steps for the Z-DNA and 5mCZ-DNA simulations, respectively. Y-axes show the densities. X-axes are in Angstroms in the top row (buckle, propel, opening) and degrees in the bottom row (shear, stretch, stagger).
(PNG)

Figure S7 Representative distributions of base pair step parameters in Z-DNA (black) and 5mCZ-DNA (red) simulations. Note that the two bimodal distributions in slide and twist correspond to GpC vs. CpG steps (see Figure 7). y-axes show the densities. x-axes are in Angstroms in the top row (shift, slide, rise) and degrees in the bottom row (tilt, roll, twist).
(PNG)

Figure S8 Representative distributions of base pair step parameters for GpC and CpG steps in Z-DNA and 5mCZ-DNA simulations. Black and green lines show the CpG steps, whereas red and blue lines show the GpC steps for the Z-DNA and 5mCZ-DNA simulations, respectively. Y-axes show the densities. X-axes are in Angstroms in the top row (shift, slide, rise) and degrees in the bottom row (tilt, roll, twist).
(PNG)

Figure S9 Representative distributions of sugar phosphate backbone torsional angles in B-DNA (black) and 5mCB-DNA (red) simulations. The x axes are in degrees.
(PNG)

Figure S10 Representative distributions of sugar pucker parameters in B-DNA (black) and 5mCB-DNA (red) simulations. The x axes are in degrees.
(PNG)

Figure S11 Representative distributions of sugar phosphate backbone torsional angles in Z-DNA (black) and 5mCZ-DNA (red) simulations. The x axes are in degrees.
(PNG)

Figure S12 Representative distributions of sugar pucker parameters in Z-DNA (black) and 5mCZ-DNA (red) simulations. The x axes are in degrees.
(PNG)

Figure S13 Time evolution of ε- ζ for individual bases in representative unmethylated B-DNA (blue) and methylated 5mCB-DNA (red) simulations for base pairs 2–4 showing the jumps between BI and BII states. Left columns are Watson strand, and right columns are Crick strand. A ε-ζ<0 indicates that the base is in the BI conformation.
(PNG)

Figure S14 Time evolution of ε- ζ for individual bases in representative B-DNA (blue) and 5mCB-DNA (red) simulations for base pairs 6–9 showing the jumps between BI and BII states. Left columns are Watson strand, and right columns are Crick strand. A ε-ζ<0 indicates that the base is in BI conformation.
(PNG)

Figure S15 Cumulative means of the fraction of BI conformations (top) and the fraction of canonical conformations (bottom) in B-DNA (black) and 5mCB-DNA (red) simulations. Solid and dashed lines indicate the two independent MD runs. Note that in one methylated B-DNA trajectory the 3′ terminal base pair breaks and reforms around 20–25 ns.
(PNG)

Figure S16 Scatter plots of α vs. γ for CpG and GpC steps of Z-DNA simulations. (A) Z-DNA GpC steps; (B) Z-DNA CpG steps; (C) 5mCZ-DNA Gp5mC steps; (D) 5mCZ-DNA 5mCpG steps. The plots are color-coded based on the density of points.
(PNG)

Table S1 Sequence-averaged conformational parameters I: Base pair parameters
(DOC)

Table S2 Sequence-averaged conformational parameters II: Base pair step parameters
(DOCX)

Table S3 Sequence-averaged conformational parameters III: Helical parameters
(DOCX)

Table S4 Sequence-averaged conformational parameters IV: Backbone torsions
(DOCX)

Table S5 Average geometric parameters from high resolution X-ray structures
(DOCX)

Table S6 Percent of sugar pucker values from the eight simulations.
(DOCX)

Table S7 Percent of α/γ conformational states for the 8 composite MD simulations.
(DOCX)

## References

[1] Maunakea AK, Chepelev I, Zhao K (2010). Epigenome mapping in normal and disease States.. Circ Res.

[2] Feng S, Jacobsen SE, Reik W (2010). Epigenetic reprogramming in plant and animal development.. Science.

[3] Sharma RP, Gavin DP, Grayson DR (2010). CpG methylation in neurons: message, memory, or mask?. Neuropsychopharmacol.

[4] Kulis M, Esteller M (2010). DNA methylation and cancer.. Adv Genet.

[5] Cooper DN, Mort M, Stenson PD, Ball EV, Chuzhanova NA (2010). Methylation-mediated deamination of 5-methylcytosine appears to give rise to mutations causing human inherited disease in CpNpG trinucleotides, as well as in CpG dinucleotides.. Hum Genomics.

[6] Rubin AF, Green P (2009). Mutation patterns in cancer genomes.. Proc Natl Acad Sci U S A.

[7] Hartmann B, Piazzola D, Lavery R (1993). BI–BII transitions in B-DNA.. Nucleic Acids Res.

[8] Heddi B, Foloppe N, Bouchemal N, Hantz E, Hartmann B (2006). Quantification of DNA BI/BII backbone states in solution. Implications for DNA overall structure and recognition.. J Am Chem Soc.

[9] Mirkin SM (2008). Discovery of alternative DNA structures: a heroic decade (1979–1989).. Front Biosci.

[10] Wells RD (2007). Non-B DNA conformations, mutagenesis and disease.. Trends Biochem Sci.

[11] Phan AT, Kuryavyi V, Patel DJ (2006). DNA architecture: from G to Z.. Curr Opin Struct Biol.

[12] Bacolla A, Wells RD (2009). Non-B DNA conformations as determinants of mutagenesis and human disease.. Mol Carcinog.

[13] Crawford JL, Kolpak FJ, Wang AH, Quigley GJ, van Boom JH (1980). The tetramer d(CpGpCpG) crystallizes as a left-handed double helix.. Proc Natl Acad Sci U S A.

[14] Kim D, Reddy S, Kim DY, Rich A, Lee S (2009). Base extrusion is found at helical junctions between right- and left-handed forms of DNA and RNA.. Nucleic Acids Res.

[15] Ha SC, Lowenhaupt K, Rich A, Kim YG, Kim KK (2005). Crystal structure of a junction between B-DNA and Z-DNA reveals two extruded bases.. Nature.

[16] Behe M, Felsenfeld G (1981). Effects of methylation on a synthetic polynucleotide: the B–Z transition in poly(dG-m5dC).poly(dG-m5dC).. Proc Natl Acad Sci U S A.

[17] Herbert A, Rich A (1996). The biology of left-handed Z-DNA.. J Biol Chem.

[18] Bae S, Kim D, Kim KK, Kim YG, Hohng S (2011). Intrinsic Z-DNA is stabilized by the conformational selection mechanism of Z-DNA-binding proteins.. J Am Chem Soc.

[19] Lee M, Kim SH, Hong SC (2010). Minute negative superhelicity is sufficient to induce the B-Z transition in the presence of low tension.. Proc Natl Acad Sci U S A.

[20] Perez A, Lankas F, Luque FJ, Orozco M (2008). Towards a molecular dynamics consensus view of B-DNA flexibility.. Nucleic Acids Res.

[21] Orozco M, Noy A, Perez A (2008). Recent advances in the study of nucleic acid flexibility by molecular dynamics.. Curr Opin Struct Biol.

[22] Lavery R, Zakrzewska K, Beveridge D, Bishop TC, Case DA (2010). A systematic molecular dynamics study of nearest-neighbor effects on base pair and base pair step conformations and fluctuations in B-DNA.. Nucleic Acids Res.

[23] Perez A, Marchan I, Svozil D, Sponer J, Cheatham TE (2007). Refinement of the AMBER force field for nucleic acids: improving the description of alpha/gamma conformers.. Biophys J.

[24] Cheatham TE, Cieplak P, Kollman PA (1999). A modified version of the Cornell et al. force field with improved sugar pucker phases and helical repeat.. J Biomol Struct Dyn.

[25] Perez A, Luque FJ, Orozco M (2007). Dynamics of B-DNA on the microsecond time scale.. J Am Chem Soc.

[26] Ponomarev SY, Thayer KM, Beveridge DL (2004). Ion motions in molecular dynamics simulations on DNA.. Proc Natl Acad Sci U S A.

[27] Rauch C, Trieb M, Wibowo FR, Wellenzohn B, Mayer E (2005). Towards an understanding of DNA recognition by the methyl-CpG binding domain 1.. J Biomol Struct Dyn.

[28] Rauch C, Trieb M, Wellenzohn B, Loferer M, Voegele A (2003). C5-methylation of cytosine in B-DNA thermodynamically and kinetically stabilizes BI.. J Am Chem Soc.

[29] Djuranovic D, Hartmann B (2004). DNA fine structure and dynamics in crystals and in solution: the impact of BI/BII backbone conformations.. Biopolymers.

[30] Heddi B, Oguey C, Lavelle C, Foloppe N, Hartmann B (2010). Intrinsic flexibility of B-DNA: the experimental TRX scale.. Nucleic Acids Res.

[31] Djuranovic D, Hartmann B (2003). Conformational characteristics and correlations in crystal structures of nucleic acid oligonucleotides: evidence for sub-states.. J Biomol Struct Dyn.

[32] Varnai P, Djuranovic D, Lavery R, Hartmann B (2002). Alpha/gamma transitions in the B-DNA backbone.. Nucleic Acids Res.

[33] Herbert A, Rich A (1999). Left-handed Z-DNA: structure and function.. Genetica.

[34] Rauch C, Pichler A, Trieb M, Wellenzohn B, Liedl KR (2005). Z-DNA's conformer substates revealed by FT-IR difference spectroscopy of nonoriented left-handed double helical poly(dG-dC).. J Biomol Struct Dyn.

[35] Srivastava HK, Chourasia M, Kumar D, Sastry GN (2011). Comparison of Computational Methods to Model DNA Minor Groove Binders.. J Chem Inf Mod.

[36] Brice AR, Dominy BN (2011). Analyzing the robustness of the MM/PBSA free energy calculation method: Application to DNA conformational transitions.. J Comput Chem.

[37] Kollman PA, Massova I, Reyes C, Kuhn B, Huo S (2000). Calculating Structures and Free Energies of Complex Molecules: Combining Molecular Mechanics and Continuum Models.. Acc Chem Res.

[38] Killian BJ, Yundenfreund Kravitz J, Gilson MK (2007). Extraction of configurational entropy from molecular simulations via an expansion approximation.. J Chem Phys.

[39] Lee J, Kim YG, Kim KK, Seok C (2010). Transition between B-DNA and Z-DNA: free energy landscape for the B-Z junction propagation.. J Phys Chem B.

[40] Peck LJ, Wang JC (1983). Energetics of B-to-Z transition in DNA.. Proc Natl Acad Sci U S A.

[41] Cheatham TE (2004). Simulation and modeling of nucleic acid structure, dynamics and interactions.. Curr Op Struct Biol.

[42] Várnai P, Zakrzewska K (2004). DNA and its counterions: a molecular dynamics study.. Nucleic Acids Res.

[43] Dixit SB, Beveridge DL, Case DA, Cheatham TE, Giudice E (2005). Molecular dynamics simulations of the 136 unique tetranucleotide sequences of DNA oligonucleotides. II: sequence context effects on the dynamical structures of the 10 unique dinucleotide steps.. Biophys J.

[44] Beveridge DL, Barreiro G, Byun KS, Case DA, Cheatham TE (2004). Molecular dynamics simulations of the 136 unique tetranucleotide sequences of DNA oligonucleotides. I. Research design and results on d(CpG) steps.. Biophys J.

[45] Bothe JR, Lowenhaupt K, Al-Hashimi HM (2011). Sequence-Specific B-DNA Flexibility Modulates Z-DNA Formation.. J Am Chem Soc.

[46] Zheng G, Lu XJ, Olson WK (2009). Web 3DNA–a web server for the analysis, reconstruction, and visualization of three-dimensional nucleic-acid structures.. Nucleic Acids Res.

[47] Pettersen EF, Goddard TD, Huang CC, Couch GS, Greenblatt DM (2004). UCSF Chimera–a visualization system for exploratory research and analysis.. J Comput Chem.

[48] Illingworth RS, Bird AP (2009). CpG islands - ‘A rough guide’.. FEBS Letters.

[49] Case DA, Darden TA, Cheatham TE, Simmerling CL, Wang J (2008). AMBER 10.

[50] Jorgensen WL, Chandrasekhar J, Madura JD, Impey RW, Klein ML (1983). Comparison of simple potential functions for simulating liquid water.. J Chem Phys.

[51] Cheatham TE, Young MA (2000). Molecular dynamics simulation of nucleic acids: Successes, limitations, and promise*.. Biopolymers.

[52] Prabhu NV, Panda M, Yang Q, Sharp KA (2008). Explicit ion, implicit water solvation for molecular dynamics of nucleic acids and highly charged molecules.. J Comput Chem.

[53] Young MA, Ravishanker G, Beveridge DL (1997). A 5-nanosecond molecular dynamics trajectory for B-DNA: analysis of structure, motions, and solvation.. Biophys J.

[54] Darden T, York D, Pedersen L (1993). Particle Mesh Ewald - an N.Log(N) Method for Ewald Sums in Large Systems.. J Chem Phys.

[55] Ryckaert J-P, Ciccotti G, Berendsen HJC (1977). Numerical integration of the cartesian equations of motion of a system with constraints: molecular dynamics of n-alkanes.. J Comp Phys.

[56] Lavery R, Moakher M, Maddocks JH, Petkeviciute D, Zakrzewska K (2009). Conformational analysis of nucleic acids revisited: Curves+.. Nucleic Acids Res.

[57] Hornik K (2011).

[58] Srinivasan J, Cheatham TE, Cieplak P, Kollman PA, Case DA (1998). Continuum Solvent Studies of the Stability of DNA, RNA, and Phosphoramidate−DNA Helices.. J Am Chem Soc.

