# Supplementary figures and images for: The Role of Methylation in the Intrinsic Dynamics of B- and Z-DNA
Source: PLoS One. 2012 Apr 17;7(4):e35558. doi: 10.1371/journal.pone.0035558 (PMC3328458; doi:10.1371/journal.pone.0035558)

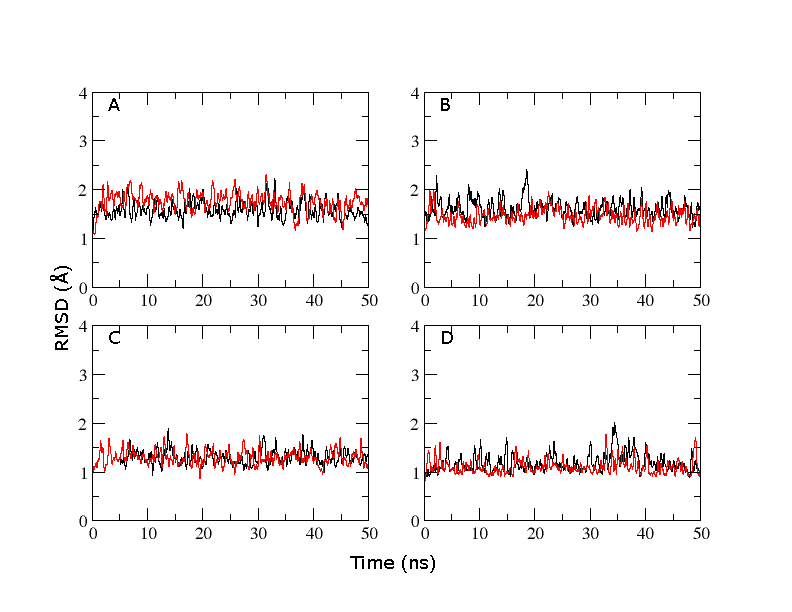

Supplement: Figure S1 — Time evolution of root mean square deviation (RMSD) of the heavy atoms from their mean positions during the simulations. A. B-DNA B. 5mCB-DNA C. Z-DNA D. 5mCZ-DNA. Black and red lines represent the two independent runs for each system. The terminal bases are excluded. (PNG) [file pone.0035558.s001.png]

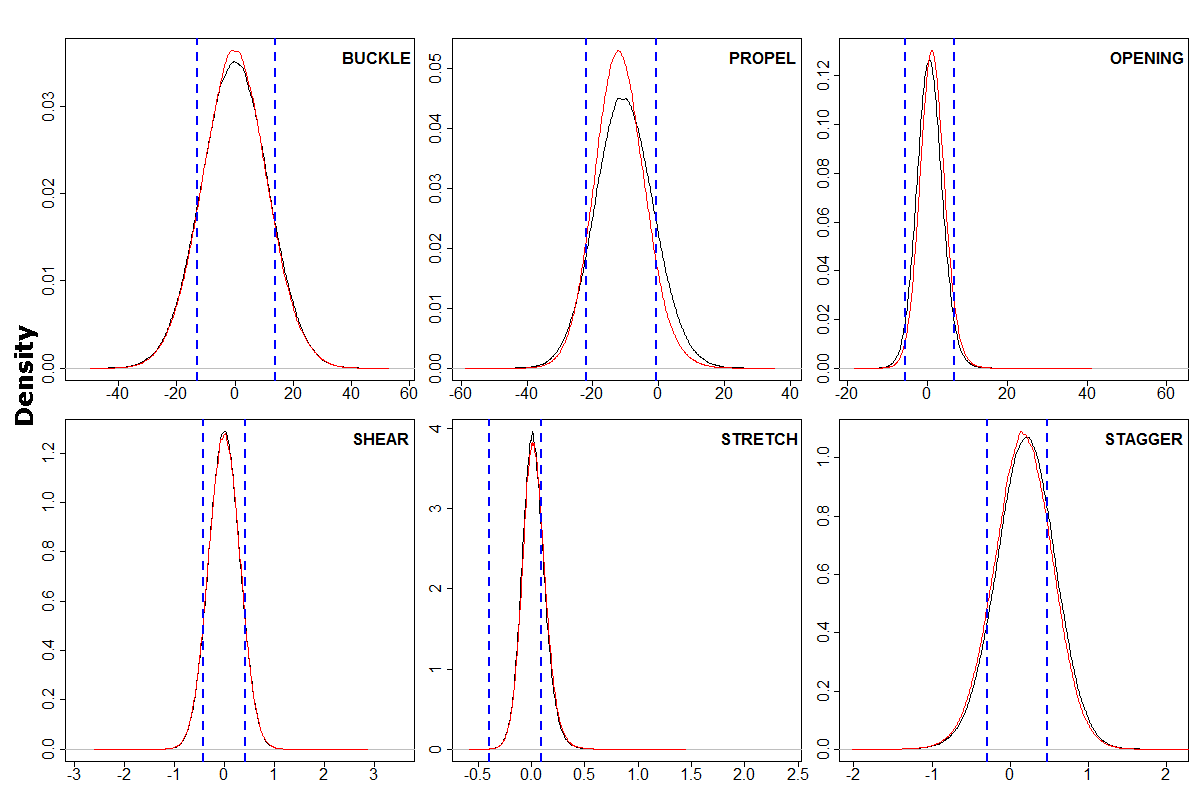

Supplement: Figure S2 — Representative distributions of base pair parameters in B-DNA (black) and 5mCB-DNA (red) simulations. Vertical dashed lines indicate the mean ± one standard deviation of the crystallographically determined values (see Table S5). Top row x-axes (buckle, propel, opening) are in degrees and bottom row x-axes (shear, stretch, stagger) are in Angstroms. Y-axes show the densities. (PNG) [file pone.0035558.s002.png]

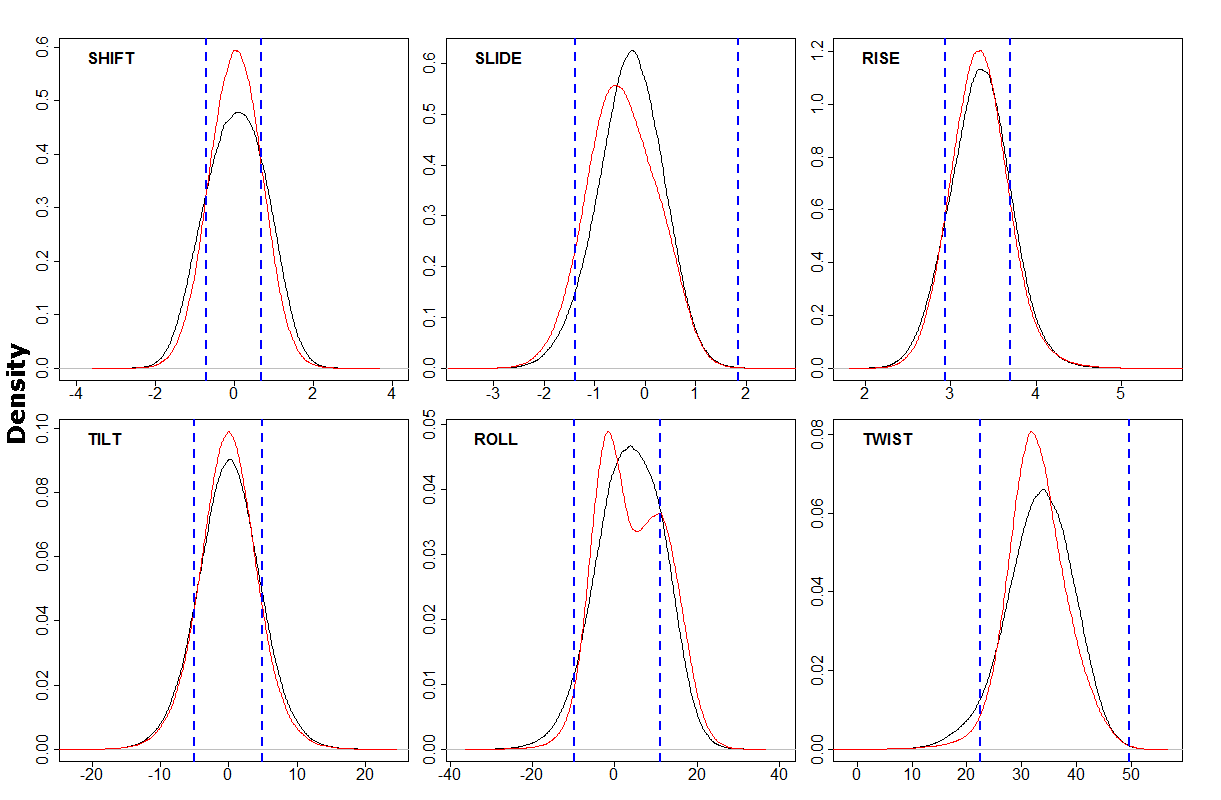

Supplement: Figure S3 — Representative distributions of base pair step parameters in B-DNA (black) and 5mCB-DNA (red) simulations. Vertical dashed lines indicate the mean ± one standard deviation of the crystallographically determined values (see Table S5). Top row x-axes (shift, slide, rise) are in Angstroms and bottom row x-axes (tilt, roll, twist) are in degrees. Y-axes show the densities. (PNG) [file pone.0035558.s003.png]

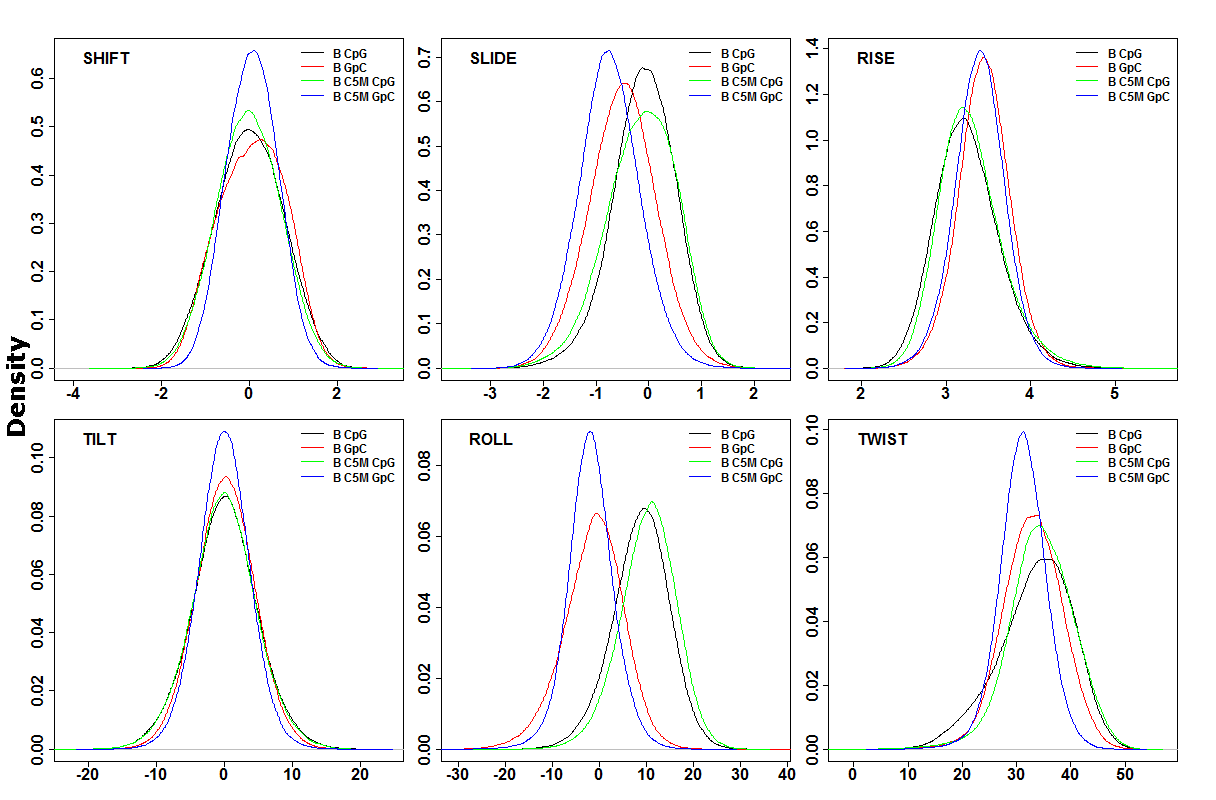

Supplement: Figure S4 — Representative distributions of base pair step parameters for GpC and CpG steps in B-DNA and 5mCB-DNA simulations. Black and green lines show the CpG steps, whereas red and blue lines show the GpC steps for the B-DNA and 5mCB-DNA simulations, respectively. Y-axes show the counts. X-axes are in Angstroms in the top row (shift, slide, rise) and degrees in the bottom row (tilt, roll, twist). (PNG) [file pone.0035558.s004.png]

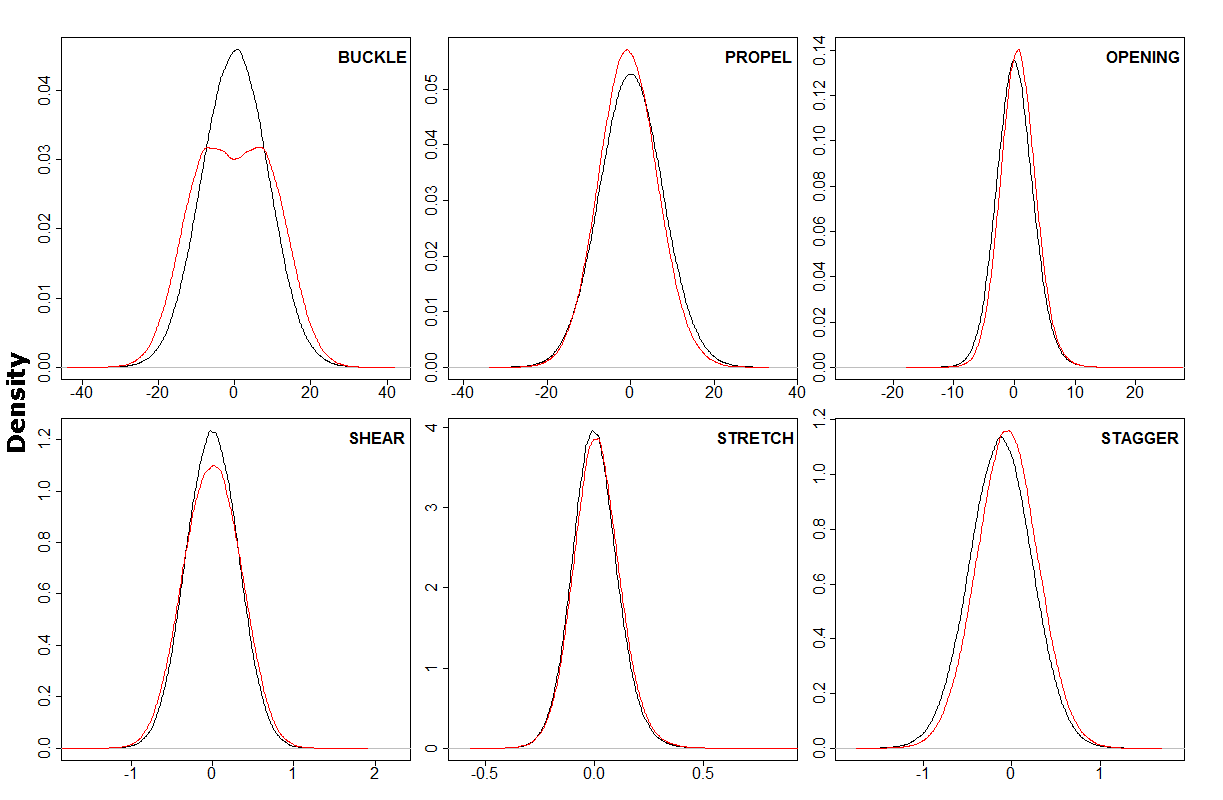

Supplement: Figure S5 — Representative distributions of base pair parameters in Z-DNA (black) and 5mCZ-DNA (red) simulations. Y-axes show the densities. X-axes are in degrees in the top row (buckle, propel, opening) and Angstroms in the bottom row (shear, stretch, stagger). (PNG) [file pone.0035558.s005.png]

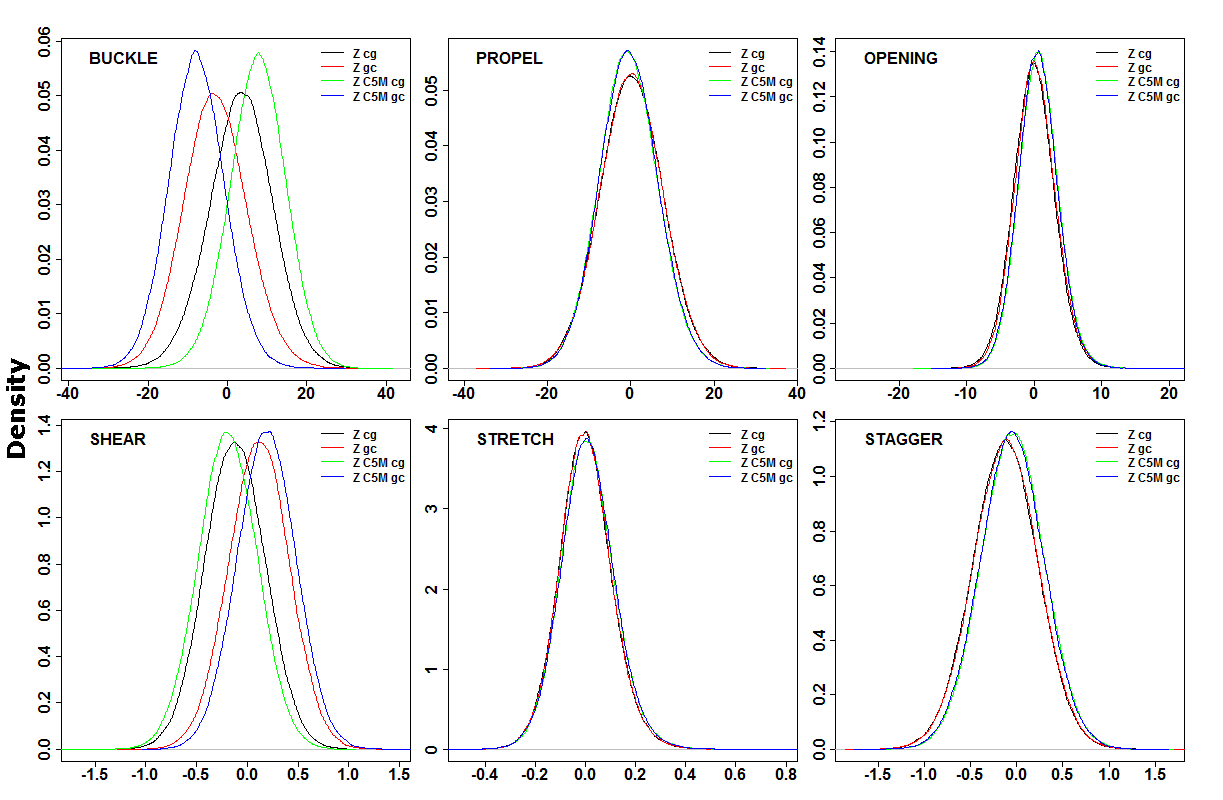

Supplement: Figure S6 — Representative distributions of base pair parameters for GpC and CpG steps in Z-DNA and 5mCZ-DNA simulations. Black and green lines show the CpG steps, whereas red and blue lines show the GpC steps for the Z-DNA and 5mCZ-DNA simulations, respectively. Y-axes show the densities. X-axes are in Angstroms in the top row (buckle, propel, opening) and degrees in the bottom row (shear, stretch, stagger). (PNG) [file pone.0035558.s006.png]

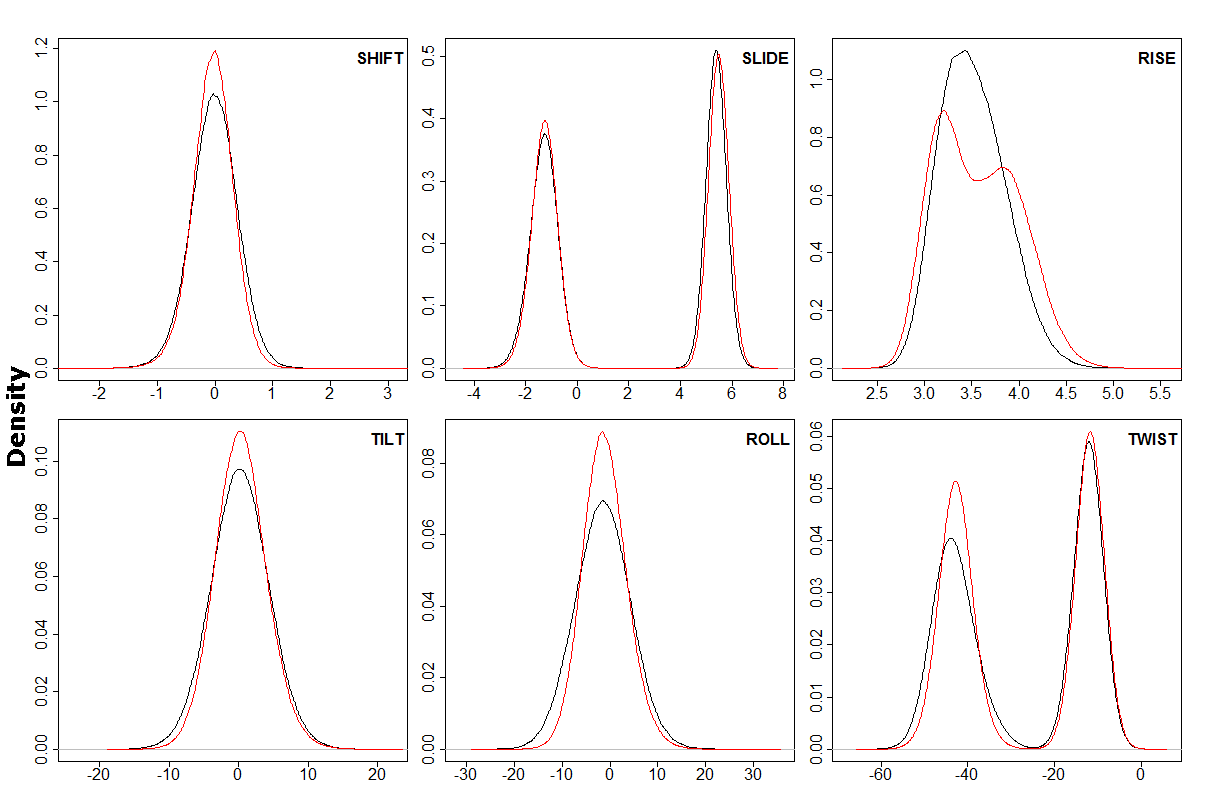

Supplement: Figure S7 — Representative distributions of base pair step parameters in Z-DNA (black) and 5mCZ-DNA (red) simulations. Note that the two bimodal distributions in slide and twist correspond to GpC vs. CpG steps (see Figure 7). y-axes show the densities. x-axes are in Angstroms in the top row (shift, slide, rise) and degrees in the bottom row (tilt, roll, twist). (PNG) [file pone.0035558.s007.png]

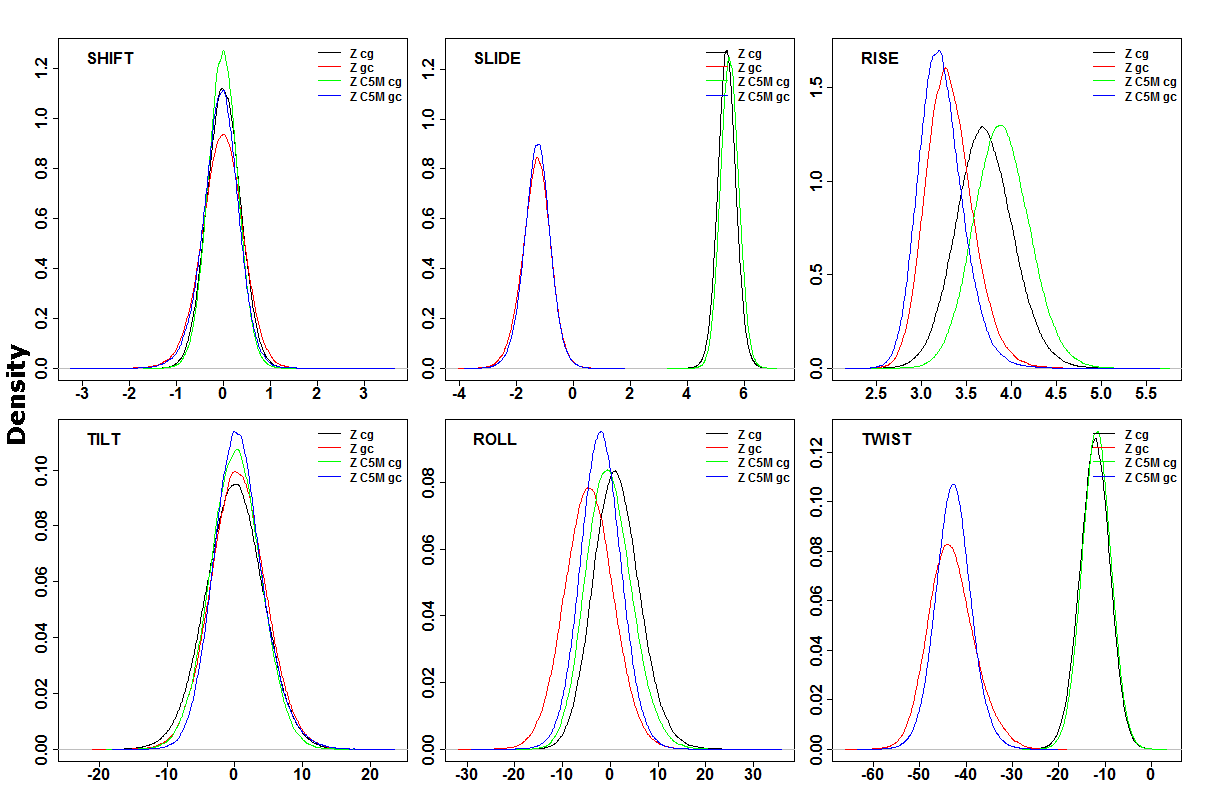

Supplement: Figure S8 — Representative distributions of base pair step parameters for GpC and CpG steps in Z-DNA and 5mCZ-DNA simulations. Black and green lines show the CpG steps, whereas red and blue lines show the GpC steps for the Z-DNA and 5mCZ-DNA simulations, respectively. Y-axes show the densities. X-axes are in Angstroms in the top row (shift, slide, rise) and degrees in the bottom row (tilt, roll, twist). (PNG) [file pone.0035558.s008.png]

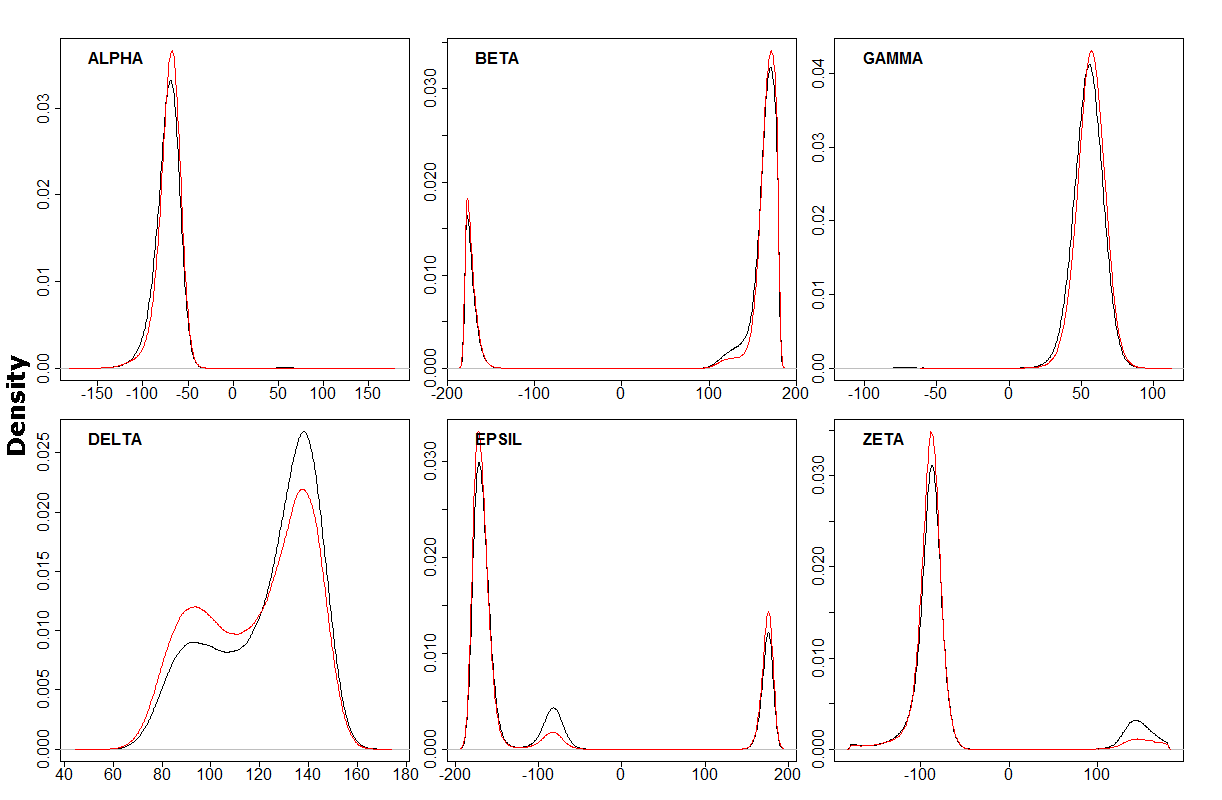

Supplement: Figure S9 — Representative distributions of sugar phosphate backbone torsional angles in B-DNA (black) and 5mCB-DNA (red) simulations. The x axes are in degrees. (PNG) [file pone.0035558.s009.png]

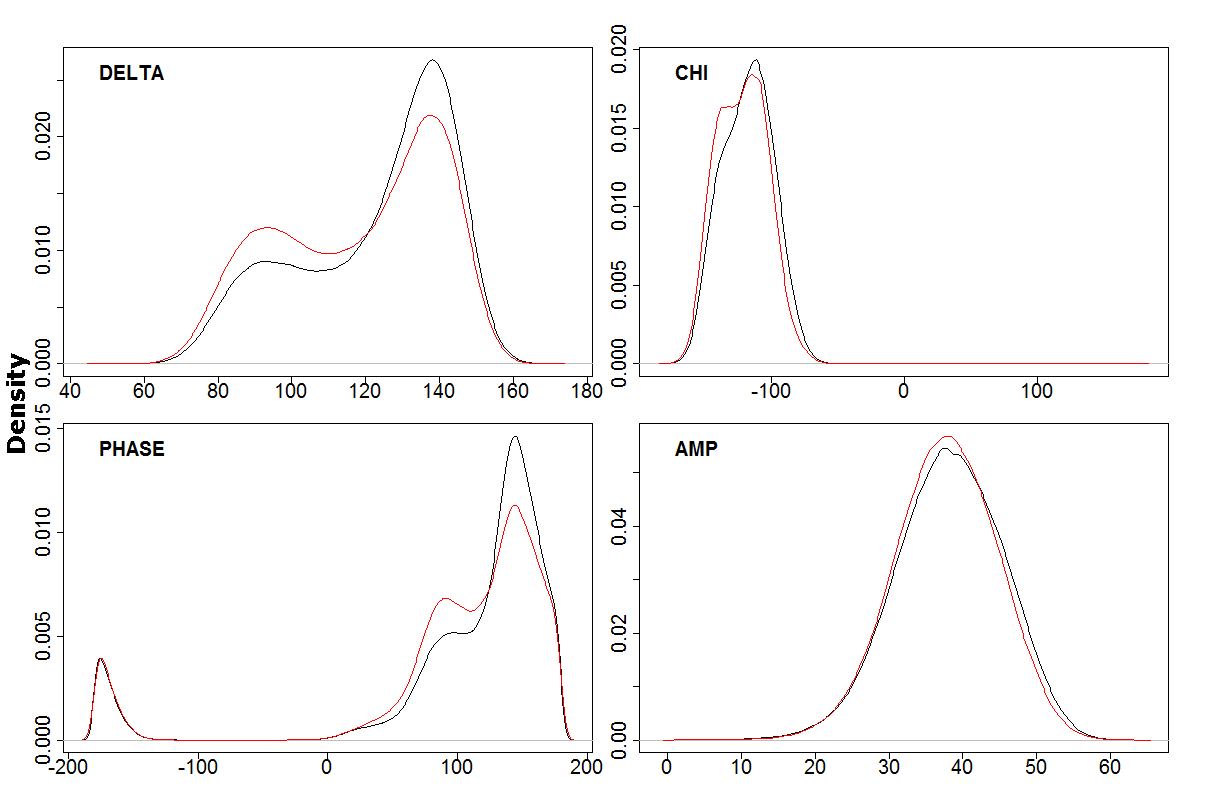

Supplement: Figure S10 — Representative distributions of sugar pucker parameters in B-DNA (black) and 5mCB-DNA (red) simulations. The x axes are in degrees. (PNG) [file pone.0035558.s010.png]

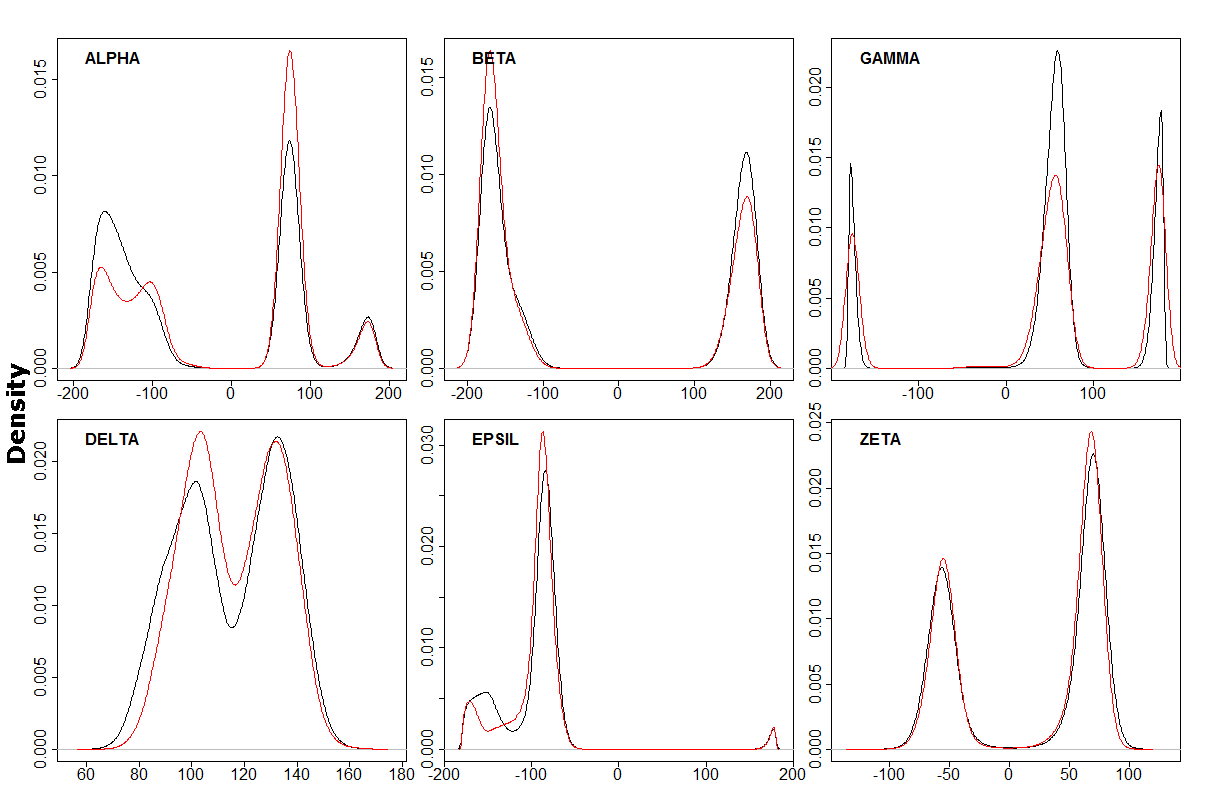

Supplement: Figure S11 — Representative distributions of sugar phosphate backbone torsional angles in Z-DNA (black) and 5mCZ-DNA (red) simulations. The x axes are in degrees. (PNG) [file pone.0035558.s011.png]

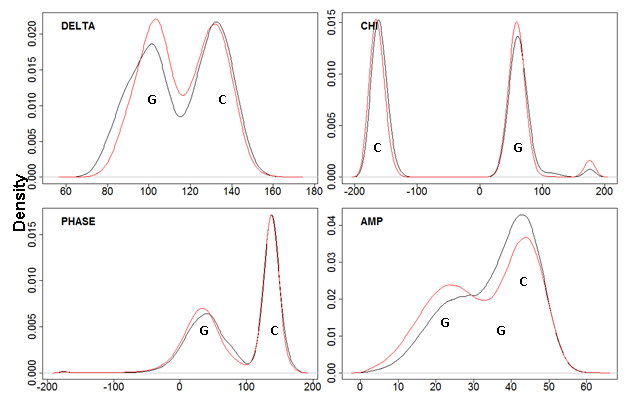

Supplement: Figure S12 — Representative distributions of sugar pucker parameters in Z-DNA (black) and 5mCZ-DNA (red) simulations. The x axes are in degrees. (PNG) [file pone.0035558.s012.png]

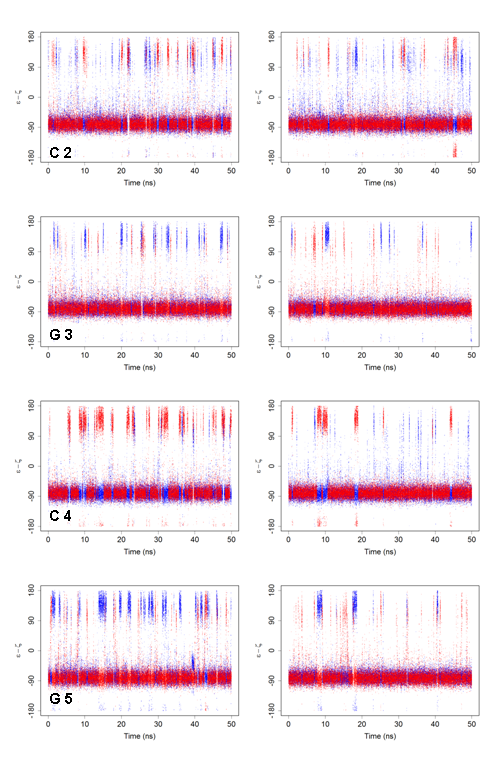

Supplement: Figure S13 — Time evolution of ε- ζ for individual bases in representative unmethylated B-DNA (blue) and methylated 5mCB-DNA (red) simulations for base pairs 2–4 showing the jumps between BI and BII states. Left columns are Watson strand, and right columns are Crick strand. A ε-ζ<0 indicates that the base is in the BI conformation. (PNG) [file pone.0035558.s013.png]

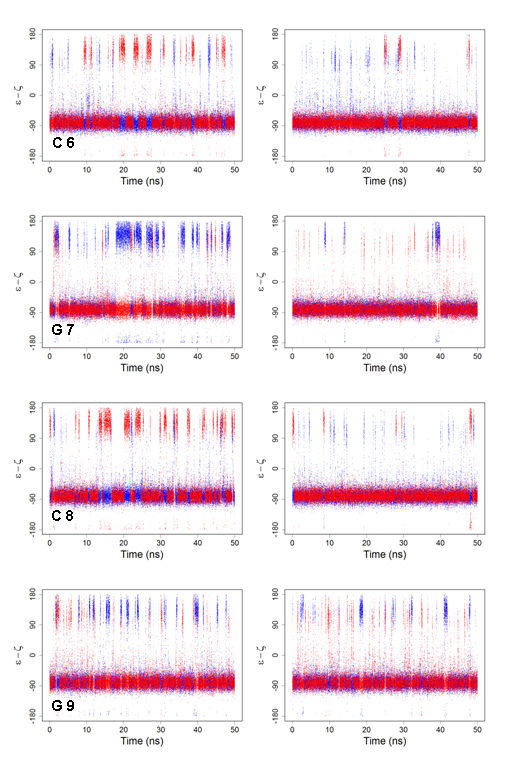

Supplement: Figure S14 — Time evolution of ε- ζ for individual bases in representative B-DNA (blue) and 5mCB-DNA (red) simulations for base pairs 6–9 showing the jumps between BI and BII states. Left columns are Watson strand, and right columns are Crick strand. A ε-ζ<0 indicates that the base is in BI conformation. (PNG) [file pone.0035558.s014.png]

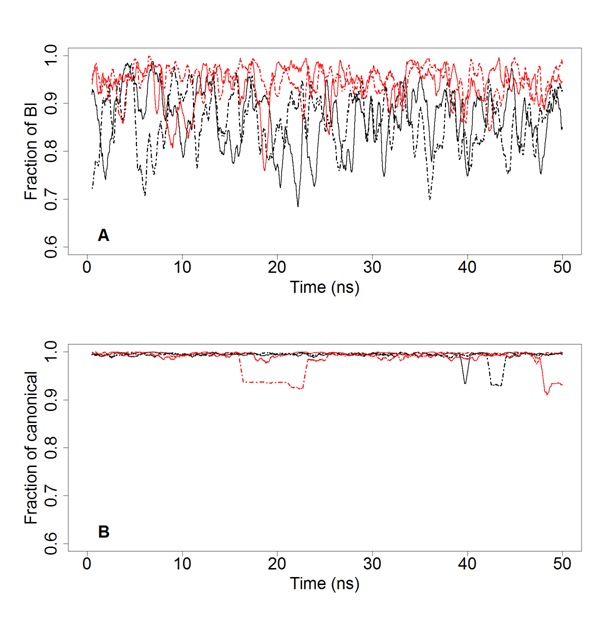

Supplement: Figure S15 — Cumulative means of the fraction of BI conformations (top) and the fraction of canonical conformations (bottom) in B-DNA (black) and 5mCB-DNA (red) simulations. Solid and dashed lines indicate the two independent MD runs. Note that in one methylated B-DNA trajectory the 3′ terminal base pair breaks and reforms around 20–25 ns. (PNG) [file pone.0035558.s015.png]

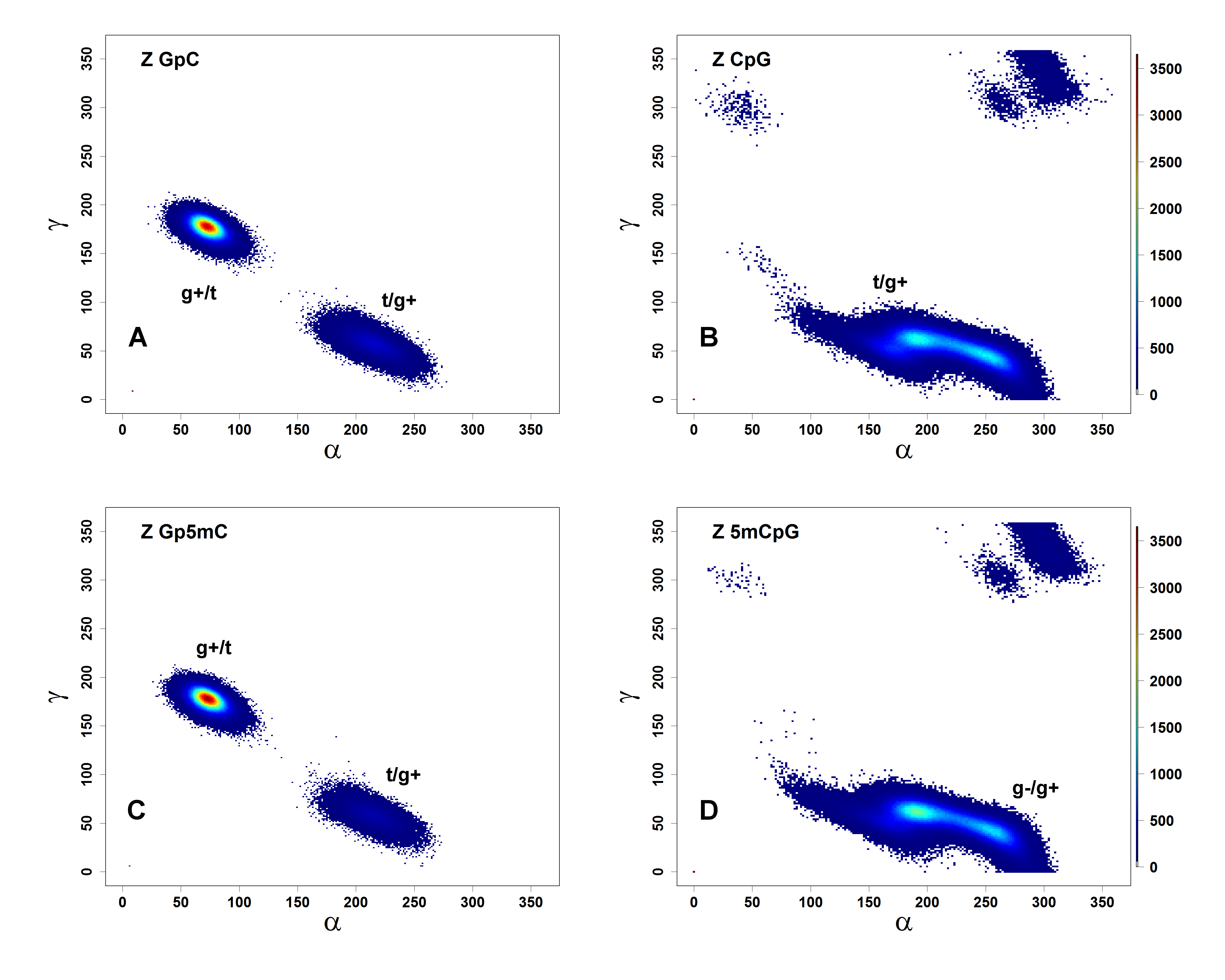

Supplement: Figure S16 — Scatter plots of α vs. γ for CpG and GpC steps of Z-DNA simulations. (A) Z-DNA GpC steps; (B) Z-DNA CpG steps; (C) 5mCZ-DNA Gp5mC steps; (D) 5mCZ-DNA 5mCpG steps. The plots are color-coded based on the density of points. (PNG) [file pone.0035558.s016.png]
