# Supplementary material for: The Role of Methylation in the Intrinsic Dynamics of B- and Z-DNA
Source: PLoS One. 2012 Apr 17;7(4):e35558. doi: 10.1371/journal.pone.0035558 (PMC3328458; doi:10.1371/journal.pone.0035558)
Supplement: Table S1 — Sequence-averaged conformational parameters I: Base pair parameters (DOC) [file pone.0035558.s017.doc]

**Table S1**. Sequence-averaged conformational parameters I: Base pair parameters

| **Parameter** | **Simulation** | **Average** | **SD** | **Range** | **Minimum** | **Maximum** |
| --- | --- | --- | --- | --- | --- | --- |
| **Shear** | **B.1** | 0.00 | 0.31 | 6.33 | -2.86 | 3.47 |
|  | **B.2** | 0.00 | 0.31 | 6.03 | -3.91 | 2.12 |
|  | **5mCB.1** | 0.00 | 0.31 | 5.33 | -2.53 | 2.80 |
|  | **5mCB.2** | 0.00 | 0.32 | 6.59 | -3.37 | 3.22 |
|  | **Z.1** | 0.00 | 0.32 | 3.85 | -1.64 | 2.21 |
|  | **Z.2** | 0.00 | 0.32 | 3.17 | -1.57 | 1.60 |
|  | **5mCZ.1** | 0.00 | 0.35 | 3.61 | -1.77 | 1.84 |
|  | **5mCZ.2** | 0.00 | 0.35 | 3.29 | -1.63 | 1.66 |
| **Stretch** | **B.1** | 0.02 | 0.11 | 2.99 | -0.60 | 2.39 |
|  | **B.2** | 0.02 | 0.11 | 2.39 | -0.49 | 1.90 |
|  | **5mCB.1** | 0.03 | 0.11 | 1.98 | -0.56 | 1.42 |
|  | **5mCB.2** | 0.03 | 0.12 | 3.98 | -0.57 | 3.41 |
|  | **Z.1** | 0.00 | 0.11 | 1.44 | -0.59 | 0.85 |
|  | **Z.2** | 0.00 | 0.11 | 1.62 | -0.61 | 1.01 |
|  | **5mCZ.1** | 0.01 | 0.11 | 1.55 | -0.55 | 1.00 |
|  | **5mCZ.2** | 0.01 | 0.11 | 1.47 | -0.55 | 0.92 |
| **Stagger** | **B.1** | 0.20 | 0.38 | 3.98 | -1.94 | 2.04 |
|  | **B.2** | 0.20 | 0.38 | 3.85 | -1.73 | 2.12 |
|  | **5mCB.1** | 0.16 | 0.37 | 4.22 | -1.94 | 2.28 |
|  | **5mCB.2** | 0.15 | 0.38 | 4.07 | -1.89 | 2.18 |
|  | **Z.1** | -0.12 | 0.36 | 3.51 | -1.77 | 1.74 |
|  | **Z.2** | -0.12 | 0.36 | 3.59 | -2.10 | 1.49 |
|  | **5mCZ.1** | -0.05 | 0.35 | 3.30 | -1.68 | 1.62 |
|  | **5mCZ.2** | -0.06 | 0.35 | 3.59 | -1.93 | 1.66 |
| **Buckle** | **B.1** | 0.0 | 11.1 | 106.60 | -51.7 | 54.9 |
|  | **B.2** | 0.1 | 11.2 | 109.80 | -57.9 | 51.9 |
|  | **5mCB.1** | 0.0 | 11.1 | 102.74 | -48.5 | 54.3 |
|  | **5mCB.2** | -0.2 | 11.1 | 100.63 | -47.9 | 52.8 |
|  | **Z.1** | 0.2 | 8.6 | 81.50 | -40.5 | 41.0 |
|  | **Z.2** | 0.0 | 8.5 | 83.70 | -41.2 | 42.5 |
|  | **5mCZ.1** | -0.1 | 10.4 | 80.95 | -41.7 | 39.8 |
|  | **5mCZ.2** | 0.0 | 10.4 | 77.44 | -37.8 | 43.3 |
| **Propeller** | **B.1** | -10.3 | 8.87 | 94.53 | -56.92 | 37.61 |
|  | **B.2** | -10.1 | 8.95 | 89.02 | -52.67 | 36.35 |
|  | **5mCB.1** | -11.7 | 7.77 | 90.64 | -57.18 | 33.46 |
|  | **5mCB.2** | -11.2 | 7.88 | 90.67 | -51.2 | 39.47 |
|  | **Z.1** | 0.19 | 7.58 | 80.58 | -39.05 | 41.53 |
|  | **Z.2** | -0.14 | 7.51 | 84.88 | -39.05 | 45.83 |
|  | **5mCZ.1** | -0.50 | 7.04 | 70.73 | -39.05 | 31.68 |
|  | **5mCZ.2** | -0.28 | 7.03 | 68.46 | -32.46 | 36 |
| **Opening** | **B.1** | 0.54 | 3.35 | 79.80 | -18.18 | 61.62 |
|  | **B.2** | 0.55 | 3.32 | 80.24 | -17.34 | 62.9 |
|  | **5mCB.1** | 1.25 | 3.23 | 58.15 | -17.81 | 40.34 |
|  | **5mCB.2** | 1.35 | 3.32 | 89.25 | -18.74 | 70.51 |
|  | **Z.1** | 0.08 | 3.08 | 52.12 | -26.76 | 25.36 |
|  | **Z.2** | 0.09 | 3.01 | 40.89 | -16.46 | 24.43 |
|  | **5mCZ.1** | 0.63 | 2.94 | 44.23 | -17.16 | 27.07 |
|  | **5mCZ.2** | 0.57 | 2.96 | 42.69 | -17.74 | 24.95 |
