# Supplementary material for: The Role of Methylation in the Intrinsic Dynamics of B- and Z-DNA
Source: PLoS One. 2012 Apr 17;7(4):e35558. doi: 10.1371/journal.pone.0035558 (PMC3328458; doi:10.1371/journal.pone.0035558)
Supplement: Table S2 — Sequence-averaged conformational parameters II: Base pair step parameters (DOCX) [file pone.0035558.s018.docx]

**Table S2**. Sequence-averaged conformational parameters II: Base pair step parameters

| **Parameter** | **Simulation** | **Average** | **SD** | **Range** | **Minimum** | **Maximum** |
| --- | --- | --- | --- | --- | --- | --- |
| **Shift** | **B.1** | 0.04 | 0.76 | 7.89 | -3.97 | 3.92 |
|  | **B.2** | 0.04 | 0.76 | 8.13 | -3.24 | 4.89 |
|  | **5mCB.1** | 0.03 | 0.66 | 7.01 | -3.46 | 3.55 |
|  | **5mCB.2** | 0.06 | 0.74 | 12.81 | -7.42 | 5.39 |
|  | **Z.1** | -0.01 | 0.40 | 5.97 | -2.95 | 3.02 |
|  | **Z.2** | -0.03 | 0.39 | 6.48 | -2.52 | 3.96 |
|  | **5mCZ.1** | -0.04 | 0.36 | 6.73 | -3.17 | 3.56 |
|  | **5mCZ.2** | -0.04 | 0.37 | 5.27 | -2.69 | 2.58 |
| **Slide** | **B.1** | -0.30 | 0.65 | 6.14 | -3.55 | 2.59 |
|  | **B.2** | -0.30 | 0.64 | 6.22 | -3.40 | 2.82 |
|  | **5mCB.1** | -0.44 | 0.70 | 6.81 | -3.76 | 3.05 |
|  | **5mCB.2** | -0.44 | 0.87 | 13.19 | -3.64 | 9.55 |
|  | **Z.1** | 2.05 | 3.37 | 11.15 | -3.92 | 7.23 |
|  | **Z.2** | 2.01 | 3.40 | 10.96 | -3.86 | 7.10 |
|  | **5mCZ.1** | 2.11 | 3.40 | 10.77 | -3.71 | 7.06 |
|  | **5mCZ.2** | 2.14 | 3.38 | 10.53 | -3.57 | 6.96 |
| **Rise** | **B.1** | 3.35 | 0.36 | 3.65 | 1.85 | 5.50 |
|  | **B.2** | 3.34 | 0.36 | 4.28 | 1.43 | 5.71 |
|  | **5mCB.1** | 3.34 | 0.35 | 3.89 | 1.88 | 5.77 |
|  | **5mCB.2** | 3.35 | 0.43 | 15.87 | -5.63 | 10.24 |
|  | **Z.1** | 3.51 | 0.35 | 3.29 | 2.23 | 5.52 |
|  | **Z.2** | 3.51 | 0.35 | 3.25 | 2.23 | 5.48 |
|  | **5mCZ.1** | 3.56 | 0.44 | 3.47 | 2.21 | 5.68 |
|  | **5mCZ.2** | 3.56 | 0.45 | 3.38 | 2.05 | 5.43 |
| **Tilt** | **B.1** | 0.09 | 4.62 | 45.51 | -22.05 | 23.46 |
|  | **B.2** | 0.04 | 4.65 | 48.06 | -25.20 | 22.86 |
|  | **5mCB.1** | -0.05 | 4.35 | 48.14 | -24.37 | 23.77 |
|  | **5mCB.2** | -0.30 | 5.85 | 178.16 | -95.16 | 83.00 |
|  | **Z.1** | 0.23 | 4.19 | 44.77 | -23.24 | 21.53 |
|  | **Z.2** | 0.23 | 4.16 | 45.94 | -20.49 | 25.45 |
|  | **5mCZ.1** | 0.37 | 3.75 | 40.84 | -18.09 | 22.75 |
|  | **5mCZ.2** | 0.47 | 3.77 | 43.39 | -20.97 | 22.42 |
| **Roll** | **B.1** | 3.75 | 8.04 | 75.32 | -36.21 | 39.11 |
|  | **B.2** | 3.89 | 7.87 | 70.88 | -35.69 | 35.19 |
|  | **5mCB.1** | 4.20 | 8.22 | 69.33 | -34.47 | 34.86 |
|  | **5mCB.2** | 4.62 | 8.61 | 149.29 | -81.76 | 67.53 |
|  | **Z.1** | -1.51 | 5.81 | 65.38 | -30.63 | 34.75 |
|  | **Z.2** | -1.66 | 5.73 | 73.39 | -37.83 | 35.56 |
|  | **5mCZ.1** | -1.08 | 4.66 | 62.88 | -28.04 | 34.84 |
|  | **5mCZ.2** | -0.78 | 4.67 | 53.82 | -26.26 | 27.56 |
| **Twist** | **B.1** | 33.19 | 6.21 | 56.39 | -0.66 | 55.73 |
|  | **B.2** | 33.07 | 6.30 | 60.56 | -5.28 | 55.28 |
|  | **5mCB.1** | 32.81 | 5.52 | 64.09 | -8.64 | 55.45 |
|  | **5mCB.2** | 32.54 | 5.81 | 199.89 | -106.03 | 93.86 |
|  | **Z.1** | -27.66 | 16.01 | 67.98 | -64.91 | 3.07 |
|  | **Z.2** | -27.95 | 16.33 | 66.83 | -64.81 | 2.02 |
|  | **5mCZ.1** | -27.30 | 15.84 | 65.08 | -62.54 | 2.54 |
|  | **5mCZ.2** | -27.29 | 15.97 | 66.79 | -64.76 | 2.03 |
