# Supplementary material for: The Role of Methylation in the Intrinsic Dynamics of B- and Z-DNA
Source: PLoS One. 2012 Apr 17;7(4):e35558. doi: 10.1371/journal.pone.0035558 (PMC3328458; doi:10.1371/journal.pone.0035558)
Supplement: Table S3 — Sequence-averaged conformational parameters III: Helical parameters (DOCX) [file pone.0035558.s019.docx]

**Table S3**. Sequence-averaged conformational parameters III: Helical parameters

| **Parameter** | **Simulation** | **Average** | **SD** | **Range** | **Minimum** | **Maximum** |
| --- | --- | --- | --- | --- | --- | --- |
| **Xdisp** | **B.1** | -1.07 | 0.88 | 8.49 | -5.63 | 2.86 |
|  | **B.2** | -1.06 | 0.86 | 8.20 | -5.20 | 3.00 |
|  | **5mCB.1** | -1.25 | 0.82 | 7.92 | -5.78 | 2.14 |
|  | **5mCB.2** | -1.42 | 0.85 | 35.92 | -20.59 | 15.33 |
|  | **Z.1** | -5.49 | 1.59 | 70.41 | -59.15 | 11.26 |
|  | **Z.2** | -5.35 | 1.60 | 78.27 | -36.49 | 41.78 |
|  | **5mCZ.1** | -4.80 | 1.52 | 54.50 | -21.57 | 32.93 |
|  | **5mCZ.2** | -4.98 | 1.53 | 41.36 | -23.46 | 17.90 |
| **Ydisp** | **B.1** | 0.02 | 0.59 | 6.27 | -3.34 | 2.93 |
|  | **B.2** | -0.03 | 0.59 | 7.41 | -3.55 | 3.86 |
|  | **5mCB.1** | 0.00 | 0.62 | 9.45 | -5.89 | 3.56 |
|  | **5mCB.2** | 0.05 | 0.66 | 31.15 | -22.21 | 8.94 |
|  | **Z.1** | -0.02 | 2.99 | 48.24 | -28.68 | 19.56 |
|  | **Z.2** | -0.02 | 3.03 | 64.27 | -29.70 | 34.57 |
|  | **5mCZ.1** | 0.00 | 2.75 | 33.88 | -17.39 | 16.49 |
|  | **5mCZ.2** | -0.03 | 2.77 | 41.76 | -26.44 | 15.32 |
| **Inclination** | **B.1** | 6.48 | 5.27 | 56.45 | -21.83 | 34.62 |
|  | **B.2** | 6.58 | 5.22 | 58.68 | -21.12 | 37.56 |
|  | **5mCB.1** | 6.59 | 4.89 | 56.82 | -23.60 | 33.22 |
|  | **5mCB.2** | 7.20 | 5.14 | 71.00 | -19.57 | 51.43 |
|  | **Z.1** | 1.60 | 5.58 | 58.65 | -30.38 | 28.27 |
|  | **Z.2** | 2.11 | 5.54 | 50.57 | -22.30 | 28.27 |
|  | **5mCZ.1** | 1.83 | 5.46 | 53.81 | -24.79 | 29.02 |
|  | **5mCZ.2** | 1.13 | 5.39 | 52.31 | -22.92 | 29.39 |
| **Tip** | **B.1** | -0.11 | 5.12 | 53.45 | -29.90 | 23.55 |
|  | **B.2** | 0.11 | 5.08 | 46.91 | -23.41 | 23.50 |
|  | **5mCB.1** | 0.10 | 5.06 | 44.22 | -22.39 | 21.83 |
|  | **5mCB.2** | -0.06 | 5.23 | 78.58 | -50.21 | 28.37 |
|  | **Z.1** | -180.19 | 4.88 | 49.50 | -204.20 | -154.70 |
|  | **Z.2** | -180.14 | 4.74 | 45.80 | -203.58 | -157.78 |
|  | **5mCZ.1** | -180.00 | 4.14 | 38.82 | -199.18 | -160.36 |
|  | **5mCZ.2** | -180.18 | 4.22 | 41.94 | -203.48 | -161.54 |
| **H-twist** | **B.1** | 33.59 | 5.96 | 55.80 | 0.60 | 56.40 |
|  | **B.2** | 33.51 | 6.05 | 59.30 | -4.20 | 55.10 |
|  | **5mCB.1** | 33.23 | 5.53 | 57.00 | -7.09 | 55.15 |
|  | **5mCB.2** | 33.06 | 5.80 | 60.70 | -137.55 | 99.14 |
|  | **Z.1** | -27.84 | 15.97 | 67.90 | -65.00 | 2.86 |
|  | **Z.2** | -28.15 | 16.31 | 68.20 | -66.10 | 2.06 |
|  | **5mCZ.1** | -27.48 | 15.79 | 64.50 | -63.02 | 2.10 |
|  | **5mCZ.2** | -27.45 | 15.92 | 71.30 | -64.31 | 1.97 |
