# Supplementary material for: The Role of Methylation in the Intrinsic Dynamics of B- and Z-DNA
Source: PLoS One. 2012 Apr 17;7(4):e35558. doi: 10.1371/journal.pone.0035558 (PMC3328458; doi:10.1371/journal.pone.0035558)
Supplement: Table S4 — Sequence-averaged conformational parameters IV: Backbone torsions (DOCX) [file pone.0035558.s020.docx]

**Table S4**. Sequence-averaged conformational parameters IV: Backbone torsions

| **Parameter** | **Simulation** | **Average** | **SD** | **Parameter** | **Simulation** | **Average** | **SD** |
| --- | --- | --- | --- | --- | --- | --- | --- |
| **α** | **B.1** | -72.84 | 13.90 | **ζ** | **B.1** | -61.85 | 79.83 |
|  | **B.2** | -72.57 | 15.14 |  | **B.2** | -60.51 | 81.21 |
|  | **5mCB.1** | -70.50 | 15.44 |  | **5mCB.1** | -78.63 | 55.17 |
|  | **5mCB.2** | -69.27 | 18.67 |  | **5mCB.2** | -80.19 | 51.03 |
|  | **Z.1** | -26.84 | 118.54 |  | **Z.1** | 18.93 | 61.25 |
|  | **Z.2** | -12.70 | 113.32 |  | **Z.2** | 20.61 | 60.02 |
|  | **5mCZ.1** | -14.60 | 114.16 |  | **5mCZ.1** | 17.81 | 60.71 |
|  | **5mCZ.2** | -14.54 | 116.38 |  | **5mCZ.2** | 18.12 | 60.48 |
| **β** | **B.1** | 95.48 | 135.22 | **χ** | **B.1** | -117.66 | 19.44 |
|  | **B.2** | 95.61 | 135.26 |  | **B.2** | -117.65 | 19.57 |
|  | **5mCB.1** | 89.35 | 141.54 |  | **5mCB.1** | -121.56 | 19.23 |
|  | **5mCB.2** | 87.75 | 143.20 |  | **5mCB.2** | -122.08 | 19.77 |
|  | **Z.1** | -22.84 | 162.51 |  | **Z.1** | 43.65 | 114.43 |
|  | **Z.2** | -27.26 | 162.76 |  | **Z.2** | -45.15 | 112.82 |
|  | **5mCZ.1** | -41.89 | 158.59 |  | **5mCZ.1** | -40.28 | 117.74 |
|  | **5mCZ.2** | -41.23 | 158.56 |  | **5mCZ.2** | -40.13 | 117.78 |
| **γ** | **B.1** | 54.81 | 10.69 | **Phase** | **B.1** | 106.03 | 84.94 |
|  | **B.2** | 54.75 | 13.21 |  | **B.2** | 106.12 | 84.38 |
|  | **5mCB.1** | 56.18 | 15.12 |  | **5mCB.1** | 99.75 | 85.11 |
|  | **5mCB.2** | 55.61 | 21.09 |  | **5mCB.2** | 99.61 | 83.66 |
|  | **Z.1** | 50.59 | 106.32 |  | **Z.1** | 88.24 | 57.98 |
|  | **Z.2** | 50.01 | 114.92 |  | **Z.2** | 84.55 | 59.99 |
|  | **5mCZ.1** | 47.77 | 115.93 |  | **5mCZ.1** | 85.53 | 58.34 |
|  | **5mCZ.2** | 47.38 | 114.51 |  | **5mCZ.2** | 86.66 | 57.76 |
| **δ** | **B.1** | 122.83 | 21.08 | **Amplitude** | **B.1** | 38.13 | 7.26 |
|  | **B.2** | 122.73 | 21.16 |  | **B.2** | 38.13 | 7.27 |
|  | **5mCB.1** | 118.67 | 21.96 |  | **5mCB.1** | 37.67 | 6.97 |
|  | **5mCB.2** | 117.91 | 22.02 |  | **5mCB.2** | 37.70 | 6.93 |
|  | **Z.1** | 115.61 | 19.56 |  | **Z.1** | 34.86 | 11.09 |
|  | **Z.2** | 115.91 | 19.28 |  | **Z.2** | 34.08 | 11.47 |
|  | **5mCZ.1** | 115.78 | 17.96 |  | **5mCZ.1** | 33.55 | 12.00 |
|  | **5mCZ.2** | 115.62 | 18.07 |  | **5mCZ.2** | 33.63 | 11.85 |
| **ε** | **B.1** | -90.97 | 133.08 |  |  |  |  |
|  | **B.2** | -91.01 | 132.53 |  |  |  |  |
|  | **5mCB.1** | -85.83 | 142.99 |  |  |  |  |
|  | **5mCB.2** | -85.54 | 143.63 |  |  |  |  |
|  | **Z.1** | -95.63 | 50.99 |  |  |  |  |
|  | **Z.2** | -92.98 | 52.66 |  |  |  |  |
|  | **5mCZ.1** | -94.55 | 45.53 |  |  |  |  |
|  | **5mCZ.2** | -94.98 | 46.47 |  |  |  |  |
