# Supplementary material for: The Role of Methylation in the Intrinsic Dynamics of B- and Z-DNA
Source: PLoS One. 2012 Apr 17;7(4):e35558. doi: 10.1371/journal.pone.0035558 (PMC3328458; doi:10.1371/journal.pone.0035558)
Supplement: Table S5 — Average geometric parameters from high resolution X-ray structures (DOCX) [file pone.0035558.s021.docx]

**Table S5.** Average geometric parameters from high resolution X-ray structures^a^

|  | **B^b^** | **B Std** | **Z^c^** | **Z Std** |
| --- | --- | --- | --- | --- |
| **SHEAR** | -0.01 | 0.32 | -0.01 | 0.63 |
| **STRETCH** | -0.18 | 0.21 | -0.17 | 0.13 |
| **STAGGER** | 0.14 | 0.25 | 0.09 | 0.19 |
| **BUCKLE** | 1.05 | 6.73 | 0.08 | 5.91 |
| **PROPELLER** | -11.35 | 6.28 | -0.51 | 3.72 |
| **OPENING** | 0.52 | 4.62 | 1.54 | 2.37 |
| **SHIFT** | -0.01 | 0.54 | -0.01 | 0.20 |
| **SLIDE** | 0.19 | 0.69 | 2.85 | 3.14 |
| **RISE** | 3.34 | 0.40 | 3.56 | 0.28 |
| **TILT** | -0.17 | 3.04 | 0.03 | 2.31 |
| **ROLL** | 0.61 | 5.50 | -2.98 | 3.27 |
| **TWIST** | 35.77 | 6.96 | -25.15 | 21.61 |
| **X_DISPLACEMENT** | 0.06 | 1.17 | -12.15 | 29.37 |
| **Y_DISPLACEMENT** | -0.02 | 0.98 | 0.41 | 8.88 |
| **HELICAL_RISE** | 3.31 | 0.41 | 3.71 | 1.92 |
| **INCLINATION** | 1.82 | 9.21 | 7.84 | 20.42 |
| **TIP** | 0.21 | 5.20 | 0.87 | 17.53 |
| **HELICAL_TWIST** | 36.32 | 6.84 | -25.84 | 21.34 |

^a^ B-DNA and Z-DNA structures with resolutions higher than 2 Å were downloaded from NDB^1^ as of 06/15/2011.

^b^B-DNA NDB code: BD0008, BD0009, BD0010, BD0017, BD0021, BD0031, BD0032, BD0043, BD0044, BD0045, BD0046, BD0053, BD0073, BD0102, BD0103, BDD001, BDF068, BDJ051, BDJ061, BDJ081, BDJB48, BDJB49, BDJB50, BDJB57, BDL001, BDL005, BDL020, BDLB13, BDLB26, BDLB84, BDLB85

^c^Z-DNA NDB code: NA0908, ZD0001, ZD0002, ZD0006, ZD0008, ZD0023, ZD0026, ZD0029, ZD0031, ZD0032, ZDD015, ZDD023, ZDF013, ZDF028, ZDF039, ZDF059, ZDF061, ZDFB03, ZDFB04, ZDFB05, ZDFB06, ZDFB10, ZDFB11, ZDFB12, ZDFB21, ZDFB25, ZDFB31, ZDFB36, ZDFB37, ZDFB41, ZDFB42, ZDFB43, ZDFB51, ZDFS33

**Reference**

(1) Berman, H. M.; Westbrook, J.; Feng, Z.; Iype, L.; Schneider, B.; Zardecki, C. *Methods Biochem Anal* **2003**, *44*, 199.
