# Supplementary material for: The Role of Methylation in the Intrinsic Dynamics of B- and Z-DNA
Source: PLoS One. 2012 Apr 17;7(4):e35558. doi: 10.1371/journal.pone.0035558 (PMC3328458; doi:10.1371/journal.pone.0035558)
Supplement: Table S6 — Percent of sugar pucker values from the eight simulations. (DOCX) [file pone.0035558.s022.docx]

**Table S6**. Percent of sugar pucker values from the eight simulations.

| **Simulation** | **C3' Endo** | **C4' Exo** | **O1' Endo** | **C1' Exo** | **C2' Endo** | **C3' Exo** | **C1' Endo** | **C2' Exo** |
| --- | --- | --- | --- | --- | --- | --- | --- | --- |
| **B.1** | 2 | 6 | 16 | 34 | 35 | 7 | 0 | 0 |
| **B.2** | 2 | 6 | 16 | 34 | 35 | 7 | 0 | 0 |
| **5mCB.1** | 2 | 7 | 21 | 32 | 30 | 8 | 0 | 0 |
| **5mCB.2** | 2 | 7 | 21 | 32 | 30 | 7 | 0 | 0 |
| **Z.1** | 18 | 20 | 6 | 30 | 20 | 1 | 0 | 4 |
| **Z.2** | 21 | 20 | 3 | 29 | 21 | 1 | 1 | 5 |
| **5mCZ.1** | 19 | 20 | 5 | 32 | 18 | 1 | 1 | 4 |
| **5mCZ.2** | 18 | 21 | 6 | 32 | 18 | 1 | 1 | 4 |
