# Supplementary material for: The Role of Methylation in the Intrinsic Dynamics of B- and Z-DNA
Source: PLoS One. 2012 Apr 17;7(4):e35558. doi: 10.1371/journal.pone.0035558 (PMC3328458; doi:10.1371/journal.pone.0035558)
Supplement: Table S7 — Percent of α/γ conformational states for the 8 composite MD simulations. (DOCX) [file pone.0035558.s023.docx]

**Table S7**. Percent of α/γ conformational states for the 8 composite MD simulations.

| **Simulation** | **g-/g-** | **g-/ t** | **g-/g+** | **t /g-** | **t /t** | **t /g+** | **g+/g-** | **g+/t** | **g+/g+** |
| --- | --- | --- | --- | --- | --- | --- | --- | --- | --- |
| **B.1** | 0 | 0 | 99 | 0 | 0 | 0 | 0 | 0 | 0 |
| **B.2** | 0 | 0 | 99 | 0 | 0 | 0 | 0 | 0 | 0 |
| **5mCB.1** | 0 | 0 | 99 | 0 | 0 | 1 | 0 | 0 | 0 |
| **5mCB.2** | 0 | 0 | 99 | 0 | 0 | 0 | 0 | 1 | 0 |
| **Z.1** | 0 | 0 | 14 | 0 | 0 | 50 | 0 | 35 | 0 |
| **Z.2** | 0 | 0 | 16 | 0 | 0 | 42 | 0 | 42 | 0 |
| **5mCZ.1** | 1 | 0 | 15 | 0 | 0 | 42 | 0 | 42 | 0 |
| **5mCZ.2** | 1 | 0 | 14 | 0 | 0 | 44 | 0 | 40 | 0 |
